# Supplementary material for: Potential effects on cardiometabolic risk factors and body composition by short message service (SMS)-guided training after recent minor stroke or transient ischaemic attack: post hoc analyses of the STROKEWALK randomised controlled trial
Source: BMJ Open. 2021 Oct 17;11(10):e054851. doi: 10.1136/bmjopen-2021-054851 (PMC8524288; doi:10.1136/bmjopen-2021-054851)
Supplement: Author's manuscript [file bmjopen-2021-054851.draft_revisions.pdf]

BMJ Open is committed to open peer review. As part of this commitment we make the peer review history of every article we publish publicly available.

When an article is published we post the peer reviewers' comments and the authors' responses online. We also post the versions of the paper that were used during peer review. These are the versions that the peer review comments apply to.

The versions of the paper that follow are the versions that were submitted during the peer review process. They are not the versions of record or the final published versions. They should not be cited or distributed as the published version of this manuscript.

BMJ Open is an open access journal and the full, final, typeset and author-corrected version of record of the manuscript is available on our site with no access controls, subscription charges or pay-per-view fees (http://bmjopen.bmj.com).

If you have any questions on BMJ Open's open peer review process please email info.bmjopen@bmj.com

BMJ Open

# Potential effects on cardio-metabolic risk factors and body composition by short message service (SMS) guided training after recent minor stroke or transient ischemic attack: Post-hoc analyses of the STROKEWALK randomized controlled trial.



| | |
|---|---|
| Journal: | *BMJ Open* |
| Manuscript ID | bmjopen-2021-054851 |
| Article Type: | Original research |
| Date Submitted by the Author: | 29-Jun-2021 |





| | |
|---|---|
| Complete List of Authors: | Vahlberg, Birgit; Uppsala Universitet Medicinska fakulteten, Department of Public Health and Caring Sciences, clinical nutrition and metabolism<br>Lundström, Erik; Uppsala Universitet Medicinska fakulteten, Department of Neuroscience<br>Eriksson, Staffan; Centre for Clinical Research, Sörmland, Uppsala University, Eskilstuna<br>Holmback, Ulf; Uppsala Universitet Medicinska fakulteten, Department of Public Health and Caring Sciences, Clinical Nutrition and Metabolism,<br>Cederholm, Tommy; Uppsala Universitet Medicinska fakulteten, Public Health/Clinical Nutrition |





| | |
|---|---|
| Keywords: | Stroke < NEUROLOGY, PREVENTIVE MEDICINE, REHABILITATION MEDICINE |



SCHOLARONE™
Manuscripts

*I, the Submitting Author has the right to grant and does grant on behalf of all authors of the Work (as defined in the below author licence), an exclusive licence and/or a non-exclusive licence for contributions from authors who are: i) UK Crown employees; ii) where BMJ has agreed a CC-BY licence shall apply, and/or iii) in accordance with the terms applicable for US Federal Government officers or employees acting as part of their official duties; on a worldwide, perpetual, irrevocable, royalty-free basis to BMJ Publishing Group Ltd ("BMJ") its licensees and where the relevant Journal is co-owned by BMJ to the co-owners of the Journal, to publish the Work in this journal and any other BMJ products and to exploit all rights, as set out in our licence.*

*The Submitting Author accepts and understands that any supply made under these terms is made by BMJ to the Submitting Author unless you are acting as an employee on behalf of your employer or a postgraduate student of an affiliated institution which is paying any applicable article publishing charge ("APC") for Open Access articles. Where the Submitting Author wishes to make the Work available on an Open Access basis (and intends to pay the relevant APC), the terms of reuse of such Open Access shall be governed by a Creative Commons licence – details of these licences and which Creative Commons licence will apply to this Work are set out in our licence referred to above.*

*Other than as permitted in any relevant BMJ Author's Self Archiving Policies, I confirm this Work has not been accepted for publication elsewhere, is not being considered for publication elsewhere and does not duplicate material already published. I confirm all authors consent to publication of this Work and authorise the granting of this licence.*

For peer review only

**Potential effects on cardio-metabolic risk factors and body composition by short message service (SMS) guided training after recent minor stroke or transient ischemic attack: Post-hoc analyses of the STROKEWALK randomized controlled trial.**

**Birgit Vahlberg**[1], **Erik Lundström**[2], **Staffan Eriksson**[3,4], **Ulf Holmbäck**[1], **Tommy Cederholm**[1].

[1]Department of Public Health and Caring Sciences, Clinical Nutrition and Metabolism, Uppsala University, Uppsala [2] Department of Neuroscience, Neurology, Uppsala University [3] Centre for Clinical Research, Sörmland, Uppsala University, Eskilstuna [4] Department of Neuroscience, Physiotherapy, Uppsala University.

All authors are from Sweden.

**Corresponding author:** Birgit Vahlberg

Address: Department of Public Health and Caring Sciences, Clinical Nutrition and Metabolism,

Uppsala University, Box 593, BMC

S-751 24 Uppsala, Sweden

Telephone: +46709583473

E-mail: Birgit.Vahlberg@pubcare.uu.se

Word count text: 2968

Word count abstract: 277

Tables: 3

Figures: 1

Clinical trial registration information: URL: http://www.clinicaltrials.gov. Unique identifier (NCT 02902367)

**Keywords:** stroke, transient ischemic attack, exercise, metabolic risk factors, body composition

For peer review only

## Abstract

**Objectives:** In post-hoc analyses evaluate whether daily mobile phone text messaging coupled with the use of a training diary and a pedometer were better than current standard treatment to improve body composition and cardio-metabolic risk markers and self-reported health at three months after stroke.

**Design:** Randomized controlled three-month intervention study with per protocol analyses.

**Setting:** University hospital in Sweden.

**Participants** Seventy-nine patients (mean (SD) age 64 (10) years, 37% female) ≥18 years with good motor function (modified Rankin Scale ≤ 2) and able to perform six-minute walking test at discharge from the hospital, were randomized to either intervention ($n$ = 40) or control group ($n$ = 39). Key exclusion criteria were subarachnoid bleeding, medical problems such as uncontrolled hypertension, severe psychiatric problems or cognitive improvements.

**Interventions:** The intervention group received standard care and daily mobile phone instructional text-messages to perform regular outdoor walking and functional leg exercises. The control group received standard care.

**Main outcome measures**: Fat mass and fat-free mass was estimated by bioelectric impedance analysis. Cardio-metabolic risk factors like blood lipids, glycated haemoglobin (HbA1c) and blood glucose were analyzed at baseline and after three months.

**Results:** Both the intervention and control group showed favorable changes in fat-free mass, fat mass as well as in most of the cholesterol related cardio-metabolic risk factors, but no between group differences were observed. Self-perceived health was good in both groups at the follow-up.


For peer review only

**Conclusions:** No clear effect of daily mobile-phone delivered training instructions were detected in body composition, cardiovascular risk factors in blood or self-perceived health after three months. Further research is needed to evaluate secondary prevention support programs in larger populations after recent stroke or transient ischemic attack.

For peer review only

Clinical trial registration information: URL: http://www.clinicaltrials.gov. Unique identifier (NCT 02902367)

*Article summary*

The results of this study should be interpreted with caution, since they are the outcome of secondary analyses of a previously reported randomized controlled trial. We observed that body composition; i.e. fat-free mass and fat mass, as well as some cardio-metabolic risk factors like various cholesterol fractions, improved slightly three months after a stroke or transient ischemic attack both in the text messaging group and in the control group. A majority of the participants reported good or fairly good health after three months. The study indicates that not a negligible amount of individuals after stroke have cardiovascular risk-factors that need to be considered for in secondary prevention.

*`Strengths and limitations of this study´*

1. First statement: The strength of this clinical trial is its prospective and randomized controlled design, with an intervention that could be performed anywhere and without extended cost for staff.
2. Second statement: The major limitation is that this is a secondary analysis using data from a previous randomized controlled trial.
3. Third statement: The post hoc study was not powered for changes in body composition and cardiovascular risk-markers in blood.

For peer review only

## Introduction

Patients with recent stroke and transient ischemic attacks are at high risk for another vascular event that might lead to permanent disability or death (1). Three out four strokes can be attributed to behavioral risk factors such as smoking, unhealthy diets and sedentary lifestyles. Transient ischemic attack and stroke share the same risk factors (1, 2). A systematic review revealed that people after stroke are highly sedentary, in particular during the acute phase (3), which may affect body composition negatively (1, 4, 5). Loss of muscle mass and increase of fat mass may contribute to insulin resistance and impaired glucose control (4). Beyond increasing muscle mass and reduction of fat mass (5, 6), physical activity and exercise may improve lipid and glucose metabolism.

Physical activity and exercising is of paramount clinical concern, and current recommendations (7) advise people to engage in regular physical exercise of moderate intensity for ≥ 30 min/day (1, 8). The optimal approach to facilitate physical activity for secondary prevention after stroke or transient ischemic attack is not clear (6, 9, 10). The use of mobile-phones in health care are gaining popularity and many apps are available (11). A simple strategy such as Short Message Service (SMS) for optimizing home-based exercising shows potential to be implemented in clinical practice (11). We have recently showed improvements in walking endurance and lower body strength after a three-month mobile-phone guided training program (12).

In this post-hoc analysis we explored whether effects from the text messaging supported training could induce improvements in body composition and cardio-metabolic risk. Thus the aims of this study in patients after recent stroke or transient ischemic attack were to investigate whether daily mobile phone text messaging coupled with the use of a training diary and a pedometer were



For peer review only - http://bmjopen.bmj.com/site/about/guidelines.xhtml

better than current standard treatment to improve body composition and cardio-metabolic risk markers and self-reported health at 3 months after stroke.

## Methods

The STROKEWALK study was a single-center, parallel-group, randomized controlled trial performed at the stroke-unit at the Uppsala University Hospital, Sweden. Ethical approval was obtained from the regional Ethical Review Board of Uppsala University Hospital, Sweden: Dnr: 2015/550, registered with ClinicalTrials.gov (NCT02902367). We used the CONSORT checklist when writing our report (13)

Design, recruitment, determination of sample size, inclusion criteria and intervention have been described in detail elsewhere (12). In summary, inclusion criteria were age 18 years or older with minor stroke (infarction or intracerebral hemorrhage; first-ever or recurrent) or transient ischemic attack; with cognition and motor capacity, e.g, walking endurance, enough to perform the 6-minute walking test (with or without walking aid).

*Procedure*

Recruitment started November 2016, and the last follow-up assessment was performed December 2018. Patients were evaluated by one physiotherapist (BV), and a research assistant performed the body composition measurements at median five days after verified event. All participants gave oral and written informed consent to be included in the study.

Study participants underwent a physical examination, laboratory tests and answered questionnaires. Demographic and comorbidity data were collected from the medical records. Blood pressure was registered as the last measurement before leaving hospital. We calculated the Charlson Comorbidity Index – an instrument that predicts 10-year survival (14) – to classify



For peer review only - http://bmjopen.bmj.com/site/about/guidelines.xhtml

comorbidity. Information on self-reported health after three months and one year were obtained from the Swedish quality Riksstroke registry. Education level was divided into university studies or not. Smokers were grouped into daily-smokers and non-smokers. Ex-smokers were categorized as non-smokers.

*Randomization*

The allocation to either intervention or control group was based on randomization, 1:1 ratio, stratified by gender. The random allocation sequence was computer generated, pre-arranged and performed with closed envelopes. One series of envelopes for men and another for women were used. The allocation procedure was performed by a research assistant within one week after the baseline measurement.

*Baseline examinations*

Examinations at baseline have been extensively described previously (10). In summary we used

- The Montreal Cognitive Assessment scale (0-30 points) to evaluate cognitive function (15), with a higher value indicating better function.
- The modified Rankin Scale (range 0 [no symptoms] to 6 [death]); where a score 2 indicate slight disability and inability to perform all previous activities (16).
- The 6-minute walking test (meters) as a sub-maximal exercise test (17). Participants walked for 6 minutes over a 30 m course and were allowed to take shorter stops to rest.
- Stress profile: The questionnaire refers to stress behaviours in everyday life and the stress profile was assessed by a four-level self-reported scale. The scale combines 20 claims with agreements expressed in terms of time urgency/impatience or easily aroused



For peer review only - http://bmjopen.bmj.com/site/about/guidelines.xhtml

irritation/hostility; for example, other people ´s mistake irritate me (18). It is scored from not at all to fully agree, 0 to 3.

- The 4-level Saltin Grimby Physical Activity Level Scale was used to measure self-reported leisure-time physical activity during the past year before stroke (19) into a) sedentary, b) some light-intensity physical activity for at least four hours/week, c) regular moderate physical activity and training, and d) regular hard physical training for competitive sports.
- The European Working group on Sarcopenia in Older People-2 definition for sarcopenia; i.e. probable sarcopenia is indicated by low muscle strength by the five-times chair-stand test > 15 seconds or handgrip strength (Jamar® dynamometer) <27 kg in men and <16 kg in women, respectively (20). Sarcopenia was confirmed by fat-free mass index (see below) < 17 $kg/m^2$ for men and 15 $kg/m^2$ for women.

*Outcome assessments for post-hoc analyses*

The study included several endpoints at three months, i.e., body composition changes in fat-free mass and fat mass by bioelectric impedance analyses, and biochemical cardio-metabolic risk factors, and nutritional status.

The fat-free mass (kg) and fat-mass (kg) were measured with the bioelectric impedance analyses device Inbody S20; Inbody, Seoul, South Korea. Measurements were performed in the supine position. Fat-free mass index and fat-mass index was calculated by dividing masses in kg by height (m) squared.

Body mass index (BMI) was calculated as body weight (kg) divided by height (m) squared. The participants were divided into categories: underweight (< 18.5 kg/ $m^2$), normal weight (18.5 to

For peer review only



For peer review only - http://bmjopen.bmj.com/site/about/guidelines.xhtml

24.9 kg/ m$^2$), overweight (25-29.9 kg/ m$^2$), obese class I (30-35 kg/ m$^2$), class II (>35 – 39.9 kg/ m$^2$) and class III (≥40 kg/ m$^2$) (1).

Non-fasting venous blood samples for baseline analyses of glycated hemoglobin (HbA1c), serum insulin-like growth factor and biochemical cardio-metabolic risk factors were obtained. The samples were frozen at -70 ° until analyses. The biochemical analyses were performed by accredited methods of the Clinical Chemistry Laboratory at the Uppsala University Hospital, Uppsala.

The cardio-metabolic biochemical risk factors total, LDL and HDL cholesterol, and triglycerides were analyzed. Baseline values of blood lipids and C-reactive protein were recorded from values obtained at admission to the emergency ward or the day after admission to the stroke-unit. A C-reactive protein level of >5 mg/l was considered to indicate ongoing inflammation.

Non-fasting blood samples at three months follow-up were collected and frozen at –70° C until analyses. The serum lipid concentrations were considered pathological when the non-HDL cholesterol (total cholesterol minus HDL cholesterol) was >2.2 mmol/L; LDL cholesterol >1.4 mmol/L and high density lipoprotein cholesterol was ≤1.2 mmol/L in women and ≤1.1 mmol/L in men (8), respectively.

Self-reported health at three months and twelve months were analyzed from data retrieved from the Riksstroke national registry. Reported answers were very good, fairly good, fairly bad, very bad health. Data were only available for the patients with stroke, not for patients with TIA.

Mortality at one year was checked by the individual's medical records.

*Text messaging intervention*



For peer review only - http://bmjopen.bmj.com/site/about/guidelines.xhtml

As previously described, the intervention was an add-on to standard care and comprised three different strategies: 1) daily text-messaging (short message service) with instructions what and how to exercise, 2) training diaries for three months and 3) pedometers for step counts registration at the first and last week of the study(12).

The daily morning text messages across the study period gave instructions on how to exercise to increase walking endurance and lower body strength. There was no possibility to text back for help or advice. The training program was initiated about one week after the patients had been randomized.

*Control intervention*

Patients in the control group were given standard post-stroke care, which usually does not include specific advice about physical activity or home exercising. The control participants had no restrictions regarding physical activity, exercise or taking part in rehabilitation services.

*Statistical analyses*

No a priori power analysis was performed in the present post-hoc study and statistical calculations were based on per protocol analysis. Categorical data are presented as percentage and descriptive data as mean and SD or median and IQR. To check for normal distribution, the Shapiro-Wilk W test and histogram viewing were used.

Significance of between group differences in physical activity at baseline were analyzed by Pearson´s chi-squared test ($\chi^2$) with Fishers exact test for nominal data or the Kruskal-Wallis test for non-normal or ordinal data. One-way ANOVA tests with Bonferroni corrections were used to analyze continuous normal-distributed data.



For peer review only - http://bmjopen.bmj.com/site/about/guidelines.xhtml

Differences in changes between baseline and the three-month follow-up between the text messaging group and the control group are presented with 95% CI and tested by non-parametric methods (Mann-Whitney U test) for ordinal or non-normally distributed variables. For all other data, parametric methods (the independent student´s t-test) were used.

Within group differences; i.e., the difference between baseline and the three-month follow-up were tested using the paired samples t-test or the Wilcoxon's signed rank test. Correlation strength was calculated by Spearman rho coefficient.

The statistical Package for the Social Sciences (SPSS), version 27, was used for the analyses (SPSS Inc, Chicago, IL, USA).The level of statistical significance was set at $P < 0.05$.

## Results

*Baseline characteristics*

Figure 1 summarizes the recruitment and participant flow in a CONSORT diagram. Out of 100 individuals that were eligible for the study, 79 were invited to participate. A total of 71 individuals completed the full study period; i.e., a retention rate of 90%. Table 1 gives baseline characteristics of the text messaging- and control groups. Fifteen percent of the included individuals had diabetes mellitus type 2. At baseline, a total of eleven individuals (15%) had a C-reactive protein level > 5 mg/L. At baseline, individuals with HbA1C ≥ 42 mmol/mol were significantly more sedentary compared to those with moderate to high physical activity, as measured with the Saltin Grimby Physical Activity Level Scale.

Table 2 displays baseline characteristics with subjects divided into groups according to the Saltin-Grimby Physical Activity Level Scale at baseline. Sedentary participants compared to those with moderate/high physical activity had an overall worse risk-factor profile; e.g. higher body mass index, HbA1c, fat-mass and a lower HDL cholesterol.



For peer review only - http://bmjopen.bmj.com/site/about/guidelines.xhtml

The body mass index range was 20.1 $kg/m^2$ to 42.8 $kg/m^2$. At baseline, fifteen percent had probable sarcopenia according to either a reduced chair-stand test or reduced handgrip strength. None had confirmed sarcopenia;, i.e. reduced lower body strength combined with low muscle mass.

*Changes of body composition and cardio-metabolic risk markers during the intervention period*

Table 3 shows that on average there was no weight change in neither the text messaging group nor in the control group at three months, whereas variability in weight changes in both groups were substantial; i.e., from –10.1 to 7.8 kg.

A positive within group effect on HDL cholesterol was seen in female participants that received daily text messages, but there were no between group effects.

Fat-free mass increased significantly in both groups, with no difference between groups (Table 3). Concurrently, fat-mass decreased in both groups, but again no difference between groups was observed.

Table 3 shows that total cholesterol, LDL and HDL cholesterol changed in beneficial directions over the three-month period irrespective of group allocation, but no significant difference between groups were found.

Only minor changes were observed for HbA1c in both groups. Serum insulin-like growth factor tended to decrease in both group, but no significant difference between groups were found.

At baseline, C-reactive protein was not associated with any of the cardiovascular risk-markers nor fat-free mass, fat mass or self-reported physical activity level.

Next we did correlation analyses at three months, and noticed that changes in fat mass was associated with changes in body mass index in the text messaging group (r = 0.40, $P = 0.021$)



For peer review only - http://bmjopen.bmj.com/site/about/guidelines.xhtml

and the control group (r = 0.56, $P$ = 0.001), respectively. In the text messaging group changes in fat-free mass was associated with changes in HDL cholesterol (r = 0.43, $P$ = 0.014).

*Self-reported health and adverse events*

One participant of the control group had passed away at one year. Data on self-reported health at three months and one year after the event could be retrieved in 62 and 50 of all participating individuals with stroke. No difference between the groups was registered; at three months 94% and one year 86% reported very good or fairly good health in the text messaging and control group, respectively.

No adverse events such as fall-related fractures, syncope or dizziness events requiring hospitalization until three months follow-up were reported.

*Patient and public involvement*

The patients were involved in how the training instructions were delivered. Otherwise no patient involvement.

## Discussion

This study is a post hoc analysis of the STROKEWALK study, where a text messaging intervention was previously reported to increase the walking capacity as well as chair-stand capacity, i.e. the primary outcomes of the study (21). However, the post hoc analyses showed that beneficial effects on body composition and some biochemical cardio-metabolic risk factors were achieved in both groups, irrespective if they received text messages or not. Thus, the positive effects on walking capacity in the intervention group did not transform to beneficial effects on body composition or the selected group of biochemical cardio-metabolic risk factors in the intervention group only.



For peer review only - http://bmjopen.bmj.com/site/about/guidelines.xhtml

It is well-known that weight reduction and regular physical exercise improve insulin sensitivity and decreases triglyceride levels (8). Aerobic physical activity such as 25-30 km brisk walking per week may increase HDL cholesterol by 0.08-0.15 mmol/L (8). Overweight and especially abdominal adiposity contributes to overall dyslipidemia and these conditions were common in this study population that also showed both excessive weight loss and weight gain after three months. Both the text messaging group and the control group increased their fat-free mass, whereas fat-mass decreased only in the text messaging intervention group. A similar positive within group effect on HDL cholesterol was observed in female participants that received daily text messages, which is in line with the positive effects on walking and lower body strength after the training intervention.

A Cochrane review concluded that there is currently limited high quality research to support lifestyle interventions targeting mortality, blood pressure, lipid profiles and insulin resistance after stroke or transient ischemic attack (22). Treatment targets for extended secondary prevention (8) for body weight could be body mass index <25 kg/m$^2$, moderately vigorous physical activity for 30-60 minutes most days, and for individuals with diabetes HbA1c <53 mmol/mol.

In recent years the importance of muscle mass for mobility, muscle strength, energy, lipid and glucose metabolism has been highlighted in rehabilitation (20, 23).  Probable sarcopenia, i.e. low muscle strength and confirmed sarcopenia, i.e. the addition of low muscle mass is gaining increasing interest as risk factors for falls and fractures. Impaired activities of daily living are associated with cardiac and respiratory disease, as well as to cognitive impairment (20). At baseline, twelve participants displayed probable sarcopenia. Thus, interventions to improve muscle function are important in a population that have suffered from stroke or transient



For peer review only - http://bmjopen.bmj.com/site/about/guidelines.xhtml

ischemic attack. Many already have a low level of physical activity before the cerebrovascular event, and after the event the activity level might become even lower if no actions are taken.

For some individuals the walking program and the number of repetitions of chair-rising might have been too low to increase muscle mass. For this purpose individualized exercise instructions with higher intensity might be needed. There are not always opportunities for supervised exercise for those with minor stroke or transient ischemic attack when discharged from the hospital. Therefore methods including tele-rehabilitation and self-training can be an alternative or complement to usual rehabilitation, especially in remote or underserved areas (24).

As this was an explorative post-hoc study, power was not calculated for the outcomes in this study. It is obvious that this post-hoc study might have been underpowered to show any differences in body composition or cardio-metabolic risk factors. The study population of the current study showed great variability in many measures, which may have implications for the necessary sample size. The effects of exercise are known to be strongest in individuals that are sedentary (25). Approximately 14% of the participants in this study were sedentary at study start. By chance nine of the eleven sedentary individuals were randomized to the control group, which might have had an impact on the results. Other potential reasons for the weak effects could be that the time period was too short to induce stronger effects, or the substantial fluctuations in weight.

In conclusion: The results of this study should be interpreted with caution, since they are the outcome of secondary analyses of a previously reported randomized controlled trial. We observed that body composition; i.e. fat-free mass and fat mass, as well as some cardio-metabolic risk factors like various cholesterol fractions, improved slightly three months after a stroke or transient ischemic attack both in the text messaging group and in the control group. A



For peer review only - http://bmjopen.bmj.com/site/about/guidelines.xhtml

majority of the participants reported good or fairly good health after three months. The study indicates that not a negligible amount of individuals after stroke have cardiovascular risk-factors that need to be considered for in secondary prevention.

*Clinical messages*

- Body composition, HDL and LDL cholesterol had improved after three months.
- Additional studies are needed in larger study populations over longer study periods to elucidate if potential cardio-metabolic beneficial effects could be achieved.
- Especially sedentary individuals need help how to increase physical activity and reduce risk of recurrent cerebrovascular events.



For peer review only - http://bmjopen.bmj.com/site/about/guidelines.xhtml

351 **Figure 1.** CONSORT diagraming. Flow chart of the study participants through the phases of the
352 original randomized controlled trial. Data are collected in Uppsala University Hospital, Sweden
353 (November 2016 - December 2018).

354 **Conflicts of interest:** None.

355 **Author contributions:** All authors were involved in the design of the paper and in writing and
356 redrafting the manuscript. BV was responsible and the guarantor for the execution of the study
357 and Uppsala Clinical Research were involved in performing the statistical analyses.

358 **Funding:** This research received no specific grant from any funding agency in the public,
359 commercial or not-for-fit sectors

360 **Acknowledgement**

361 We thank research assistant Mia Berglund for the co-operation in the execution of the study. We
362 also thank Marina Lahti (www.intime.se) for assisting with sending the text messages.

363 The dataset used and analyzed for this study will be available from the corresponding author
364 upon a reasonable request.

365 **REFERENCES**

366 1. Virani SS, Alonso A, Benjamin EJ, Bittencourt MS, Callaway CW, Carson AP, et al.
367 Heart Disease and Stroke Statistics-2020 Update: A Report From the American Heart
368 Association. Circulation. 2020;141(9):e139-e596.

369 2. RIKSstroke. RIKSstroke, the Swedish Stroke Register. availble
370 from:htpp://www.riksstroke.org; (cited January, 2021); .



For peer review only - http://bmjopen.bmj.com/site/about/guidelines.xhtml

3. Fini NA, Holland AE, Keating J, Simek J, Bernhardt J. How Physically Active Are People Following Stroke? Systematic Review and Quantitative Synthesis. Phys Ther. 2017;97(7):707-17.

4. English C, McLennan H, Thoirs K, Coates A, Bernhardt J. Loss of skeletal muscle mass after stroke: a systematic review. Int J Stroke. 2010;5(5):395-402.

5. English C, Thoirs K, Coates A, Ryan A, Bernhardt J. Changes in fat mass in stroke survivors: a systematic review. Int J Stroke. 2012;7(6):491-8.

6. D'Isabella NT, Shkredova DA, Richardson JA, Tang A. Effects of exercise on cardiovascular risk factors following stroke or transient ischemic attack: a systematic review and meta-analysis. Clin Rehabil. 2017;31(12):1561-72.

7. World, Health, Organization. World Health Organization. WHO guidelines on physical activity and sedentary behaviour 2020. Geneva, Switzerland: World Health Organization, 2020. https://www.who.int/publications/i/item/9789240015128 (23 April 2021).

8. Mach F, Baigent C, Catapano AL, Koskinas KC, Casula M, Badimon L, et al. 2019 ESC/EAS Guidelines for the management of dyslipidaemias: lipid modification to reduce cardiovascular risk. Eur Heart J. 2020;41(1):111-88.

9. Heron N, Kee F, Cardwell C, Tully MA, Donnelly M, Cupples ME. Secondary prevention lifestyle interventions initiated within 90 days after TIA or 'minor' stroke: a systematic review and meta-analysis of rehabilitation programmes. Br J Gen Pract. 2017;67(654):e57-e66.

10. Moore SA, Hallsworth K, Plötz T, Ford GA, Rochester L, Trenell MI. Physical activity, sedentary behaviour and metabolic control following stroke: a cross-sectional and longitudinal study. PLoS One. 2013;8(1):e55263.



For peer review only

For peer review only - http://bmjopen.bmj.com/site/about/guidelines.xhtml

11. Gandhi S, Chen S, Hong L, Sun K, Gong E, Li C, et al. Effect of Mobile Health Interventions on the Secondary Prevention of Cardiovascular Disease: Systematic Review and Meta-analysis. Can J Cardiol. 2017;33(2):219-31.

12. Vahlberg B, Lundström E, Eriksson S, Holmbäck U, Cederholm T. Effects on walking performance and lower body strength by short message service guided training after stroke or transient ischemic attack (The STROKEWALK Study): a randomized controlled trial. Clin Rehabil. 2021;35(2):276-87.

13. Schulz KF, Altman DG, Moher D, Group C. CONSORT 2010 Statement: Updated guidelines for reporting parallel group randomised trials. J Clin Epidemiol. 2010;63(8):834-40.

14. Goldstein LB, Samsa GP, Matchar DB, Horner RD. Charlson Index comorbidity adjustment for ischemic stroke outcome studies. Stroke. 2004;35(8):1941-5.

15. Nasreddine ZS, Phillips N, Chertkow H. Normative data for the Montreal Cognitive Assessment (MoCA) in a population-based sample. Neurology. 2012;78(10):765-6; author reply 6.

16. Banks JL, Marotta CA. Outcomes validity and reliability of the modified Rankin scale: implications for stroke clinical trials: a literature review and synthesis. Stroke. 2007;38(3):1091-6.

17. ATS statement: guidelines for the six-minute walk test. Am J Respir Crit Care Med. 2002;166(1):111-7.

18. Claesson M, Birgander LS, Lindahl B, Nasic S, Aström M, Asplund K, et al. Women's hearts--stress management for women with ischemic heart disease: explanatory analyses of a randomized controlled trial. J Cardiopulm Rehabil. 2005;25(2):93-102.

For peer review only



For peer review only - http://bmjopen.bmj.com/site/about/guidelines.xhtml

19. Rödjer L, Jonsdottir IH, Rosengren A, Björck L, Grimby G, Thelle DS, et al. Self-reported leisure time physical activity: a useful assessment tool in everyday health care. BMC Public Health. 2012;12:693.

20. Cruz-Jentoft AJ, Bahat G, Bauer J, Boirie Y, Bruyère O, Cederholm T, et al. Sarcopenia: revised European consensus on definition and diagnosis. Age Ageing. 2019;48(4):601.

21. Vahlberg B LE, Eriksson S, Holmbäck U, Cederholm T. Effects on walking performance and lower body strength by short message service guided training after stroke or transient ischemic attack (The STROKEWALK Study): a randomized controlled trialb>. Clinical Rehabilitation. 2020, September 18;Online.

22. Mackay-Lyons M, Thornton M, Ruggles T, Che M. Non-pharmacological interventions for preventing secondary vascular events after stroke or transient ischemic attack. Cochrane Database Syst Rev. 2013;3:CD008656.

23. Biolo G, Cederholm T, Muscaritoli M. Muscle contractile and metabolic dysfunction is a common feature of sarcopenia of aging and chronic diseases: From sarcopenic obesity to cachexia. Clin Nutr. 2014.

24. Tchero H, Tabue Teguo M, Lannuzel A, Rusch E. Telerehabilitation for Stroke Survivors: Systematic Review and Meta-Analysis. J Med Internet Res. 2018;20(10):e10867.

25. Virani SS, Alonso A, Aparicio HJ, Benjamin EJ, Bittencourt MS, Callaway CW, et al. Heart Disease and Stroke Statistics-2021 Update: A Report From the American Heart Association. Circulation. 2021;143(8):e254-e743.



For peer review only - http://bmjopen.bmj.com/site/about/guidelines.xhtml

Table.1 Baseline characteristics of individuals allocated to the text messaging or control group. Data given as n (%) if not otherwise stated.

| | **Text messaging (N=40)** | **Control group (N=39)** |
|---|---|---|
| Sex, male | 26 (67) | 24 (60) |
| Age (years), mean (SD) | 63.9 (10) | 63.9 (10) |
| **Type of stroke,** | | |
| Cerebral infarction | 29 (72) | 28 (72) |
| Intra-cerebral hemorrhage | 4 (10) | 5 (13) |
| Transient Ischemic Attack | 7 (18) | 6 (15) |
| **Lifestyle factors** | | |
| Saltin Grimby Physical Activity Level Scale, score | | |
| Sedentary | 2 (5) | 9 (23) |
| Light physical activity | 29 (73) | 24 (62) |
| Moderate/high physical activity | 9 (22) | 6 (15) |
| Body Mass Index (kg/$m^2$ ), mean SD | 27.3 (4.2) | 27.7 (4.9) |
| Normal weight <25 | 12 (30.0) | 12 (30.8) |
| Overweight, 25-29.9 | 17(42.5) | 17 (43.6) |
| Obese, class I 30-35 | 9 (22.5) | 6 (15.4) |
| Obese, class II >35–39.9 | 2 (5.0) | 3 (7.7) |
| Obese, class III ≥40 | 0 | 1 (2.6) |

For peer review only



For peer review only - http://bmjopen.bmj.com/site/about/guidelines.xhtml

| | | |
|---|---|---|
| Stress profile, score, | | |
| <25 (not stressed) | 27 (68) | 25(64) |
| 25-34 (sometimes stressed) | 11(27) | 7(18) |
| ≥35 (highly stressed) | 2 (5) | 7 (18) |
| Diabetes mellitus type 2 | 5 (12) | 7 (18) |
| Glycated hemoglobin (HbA1C), mmol/mol, mean(SD) | 37.6 (7.6) | 38.4 (7.6) |
| Non diabetic | 36.4 (4.2) | 35.3 (3.4) |
| Systolic blood pressure, mean(SD) | 129.1 (14.5) | 132.2 (18.5) |
| >140 | 4 (10) | 11 (30) |
| Diastolic blood pressure, mean(SD) | 75.1 (11.4) | 77.0 (10.7) |
| >90 | 2 (5) | 3 (8) |

For peer review only



For peer review only - http://bmjopen.bmj.com/site/about/guidelines.xhtml

For peer review only



For peer review only - http://bmjopen.bmj.com/site/about/guidelines.xhtml

Table 2. Baseline characteristics of individuals with mild stroke or transient ischemic attack at baseline (n=79) related to self-reported physical activity with Saltin Grimby Physical Activity Level Scale.

| | All | Sedentary | Light physical activity | Moderate/high physical activity | P-value |
|---|---|---|---|---|---|
| n (%) | 79 (100) | 11 (14) | 53 (68) | 15 (19) | |
| Women, n (%) | 29 (37) | 2 (18) | 24 (45) | 3 (20) | |
| Age, yrs , mean (SD) | 63.9 (10.4) | 61.8 (9.0) | 64.0 (11.3) | 64.9 (8.0) | 0.75 |
| Diabetes mellitus, yes n (%) | 12 (15) | 3 (27) | 8 (15) | 1 (7) | 0.45 |
| University studies, yes n (%) | 40 (62) | 5 (46) | 26 (49) | 9 (60) | 0.79 |
| Non-smoker, n (%) | 71 (90) | 8 (73) | 48 (91) | 15 (100) | 0.087 |
| High intake fruit and vegetables, n (%) | 55 (70) | 6 (54) | 36 (69) | 13 (93) | 0.08 |
| Body weight, kg, median (IQR) | 80.90 (25.1) | 97.80 (28.60) | 80.90 (22.90) | 77.3 (11.40) | 0.027 |
| Body Mass Index, kg/m$^2$, median (IQR) | 26.90 (5.70) | 30.90 (6.50) | 27.0 (5.15) | 24.90 (2.4) | 0.016 |
| Fat-free mass, kg, mean (SD) | 57.61 (11.10) | 64.18 (10.86) | 55.72 (11.31) | 58.92 (9.45) | 0.08 |
| Fat-free mass index, kg/m$^2$, mean (SD) | 18.91 (2.14) | 20.0 (2.24) | 18.75 (2.16) | 18.64 (1.92) | 0.20 |
| Fat-free mass percent, mean (SD) | 70 (10) | 68 (10) | 69 (9.8) | 74 (9.9) | 0.21 |
| Fat-mass, kg, mean (SD) | 25.10 (10.9) | 32.0 (13.0) | 24.92 (10.35) | 18.96 (8.47) | 0.042 |
| Fat- mass index, kg/m$^2$, mean (SD) | 8.40 (3.69) | 10.0 (4.13) | 8.58 (3.63) | 6.79 (3.13) | 0.087 |
| Fat- mass percent, mean (SD) | 30 (10) | 32 (10) | 30 (9.8) | 26 (9.8) | 0.20 |

For peer review only

| | | | | | |
|---|---|---|---|---|---|
| Systolic blood pressure, (mm Hg), mean (SD) | 131 (16.6) | 137 (18.9) | 129 (15.6) | 132 (17.7) | 0.29 |
| Diastolic blood pressure, (mm Hg), median(IQR) | 75 (15) | 80 (10) | 70 (10) | 85 (20) | 0.13 |
| P - Cholesterol, (mmol/l), mean (SD) | 5.13 (1.20) | 4.52 (1.17) | 5.20 (1.19) | 5.33 (1.19) | 0.18 |
| P – LDL cholesterol, (mmol/l), median (IQR) | 3.20 (1.78) | 2.60 (1.50) | 3.20 (1.80) | 3.60 (2.13) | 0.61 |
| P – HDL cholesterol, (mmol/l), median (IQR) | 1.30 (0.50) | 0.99 (0.37) | 1.30 (0.60) | 1.35 (0.42) | 0.018 |
| Women, mean (SD), | 1.48 (0.42) | 1.11 (0.27) | 1.51 (0.44) | 1.55 (0.07) | 0.45 |
| Men, median (IQR) | 1.20 (0.43) | 0.99 (0.33) | 1.20 (0.43) | 1.25 (0.45) | 0.89 |
| P – LDL/HDL ratio, (mmol/L), mean (SD) | 2.57 (1.05) | 2.86 (1.11) | 2.46 (1.04) | 2.71 (1.07) | 0.45 |
| P - Triglycerides, (mmol/l), median (IQR) | 1.22 (0.62) | 1.49 (0.45) | 1.23 (0.64) | 1.11 (0.91) | 0.70 |
| P - Glucose, (mmol/l), median (IQR) | 6.90 (1.70) | 7.60 (2.0) | 6.65 (1.60) | 6.90 (1.70) | 0.55 |
| P - HbA1c, (mmol/mol), median (IQR) | 36.0 (5.0) | 39.0 (26.0) | 35.0 (6.0) | 35.0 (5.0) | 0.053 |
| P - C-reactive protein, (mg/L), median (IQR) | 1.70 (2.3) | 1.40 (1.63) | 2.00 (2.44) | 1.10 (3.15) | 0.51 |
| S - Insulin-like growth factor 1 (µ/L), mean (SD) | 161.8 (41.0) | 189.9 (57.0) | 156.7 (36.4) | 160.8 (40.2) | 0.11 |

Significance of between group differences was analyzed by Pearsons chi-squared test with Fishers exact test for nominal data or the Kruskal-Wallis test for non-normal or ordinal data and the one-way ANOVA test with Bonferroni correction for continuous normal distributed data. The significance level was set at $P < 0.05$.

For peer review only

Table 3. Baseline and follow-up outcome of cardio-metabolic risk factors and body composition by group, analyzed as per protocol. Data are presented as means (SD) or median (IQR).

| | | Text messaging group | | | | Control group | | | | |
|---|---|---|---|---|---|---|---|---|---|---|
| | n | Baseline | 3 month | *P* | n | Baseline | 3 month | *P* | Mean/median difference (95%CI) | P-value |
| Women | 16 | | | | 13 | | | | | |
| Body weight, kg, median(IQR) | 33 | 81.9 (15.2) | 82.4 (15.2) | 0.28 | 31 | 83.3(30.1) | 83.7 (17.7) | 0.43 | -0.2(-1.40 to 0.8) | 0.45 |
| Body Mass Index, kg/m² median(IQR) | 33 | 26.6 (6.0) | 26.36 (6.0) | 0.69 | 31 | 27.30 (5.9) | 26.36 (6.0) | 0.98 | -0.026(-0.47 to 0.36) | 0.36 |
| Fat-free mass, kg mean(SD) | 32 | 57.2 (9.4) | 59.0 (10.3) | 0.001 | 31 | 58.65 (12.9) | 59.86 (13.1) | 0.005 | -0.62(-1.9 to 0.70) | 0.35 |
| Fat-free mass%, mean(SD) | 32 | 71 (10) | 72 (10) | 0.014 | 31 | 71(10) | 72(10) | 0.029 | -0.006(-0.21 to 0.009) | 0.43 |
| Fat-free mass index, kg/m², mean(SD) | 32 | 18.93 (1.9) | 19.52 (2.0) | 0.001 | 31 | 19.00 (2.4) | 19.41 (2.5) | 0.004 | -0.18(-0.6 to 0.24) | 0.39 |
| Fat mass, kg, mean(SD) | 32 | 24.69 (11.5) | 23.39 (11.2) | 0.029 | 31 | 24.65 (10.9) | 23.89 (10.0) | 0.123 | 0.74(-0.95 to 2.0) | 0.48 |
| Fat mass%, mean(SD) | 32 | 29.3 (10) | 27.6 (10) | 0.012 | 31 | 29.1 (10) | 28.2 (9.6) | 0.041 | 0.007(-0.008 to 0.022) | 0.33 |

For peer review only

| | | | | | | | | | | |
|---|---|---|---|---|---|---|---|---|---|---|
| Fat mass index, kg/m$^2$, mean (SD) | 32 | 8.30 (3.9) | 7.89 (3.8) | 0.036 | 31 | 8.19 (3.7) | 7.93 (3.4) | 0.103 | -0.16(-0.64 to 0.33) | 0.52 |
| P-Cholesterol,(mmol/l) | 35 | 5.13 (1.19) | 4.47 (1.0) | 0.006 | 32 | 5.11 (1.23) | 4.09 (1.15) | <0.001 | -0.36(-0.99 to 0.26) | 0.25 |
| P-non- HDL cholesterol (mmol/L), mean(SD) | 34 | 3.83 (1.16) | 2.95 (1.00) | <0.001 | 32 | 3.83 (1.0) | 2.65 (0.95) | <0.001 | -0.29(-0.89 to 0.31) | 0.34 |
| P-LDL cholesterol, (mmol/l), mean(SD) | 34 | 3.17 (1.15) | 2.37 (0.88) | <0.001 | 33 | 3.13 (1.0) | 2.37 (0.80) | <0.001 | -0.30(-0.83 to 0.24) | 0.28 |
| P-HDL cholesterol. (mmol/l), mean(SD) | 34 | 1.32 (0.33) | 1.53 (0.41) | <0.001 | 33 | 1.30 (0.48) | 1.45 (0.50) | <0.001 | -0.03 (-0.13 to 0.07) | 0.55 |
| Women | 13 | 1.42 (0.37) | 1.69 (0.44) | 0.001 | 10 | 1.56 (0.57) | 1.61 (0.58) | 0.24 | -0.16 (-0.35 to 0.03) | 0.10 |
| Men | 21 | 1.26 (0.30) | 1.43 (0.37) | 0.003 | 23 | 1.18 (0.40) | 1.38 (0.50) | <0.001 | 0.037(-0.08 to 0.15) | 0.54 |
| P-LDL/HDL cholesterol, ratio, mean(SD) | 34 | 2.54 (1.00) | 1.66 (0.72) | <0.001 | 32 | 2.64 (1.05) | 1.53 (0.74) | <0.001 | -0.23 (-0.67 to 0.21) | 0.29 |
| Glycated hemoglobin, (mmol/mol), median (IQR) | 35 | 35.5 (5.0) | 37.0 (5.8) | 0.067 | 32 | 36.0 (5.0) | 37.0 (5.0) | 0.11 | 0(-1 to 1) | 0.81 |
| S-Insulin-like growth factor-1 (μ/L), mean (SD) | 29 | 167.9 (45.2) | 152.8 (52.8) | 0.061 | 33 | 157.6 (37.6) | 149.6 (41.4) | 0.086 | 7.0(-10.36 to 24.44) | 0.42 |

For peer review only

459 All within group values are presented as mean (SD). Significance of between group differences in glycated hemoglobin, weight and body mass index was analyzed by the Mann-Whitney U test. The median estimate was calculated using the Hodges-Lehmann test. Within-group differences for those variables are analyzed with Wilcoxon Signed Ranks Test.

462 Significance of between group differences in all variables except for glycated hemoglobin, weight and body mass index was analyzed by the independent samples T-test. Within group differences was analyzed by the paired samples t-test. CI indicates confidence interval. The significance level was set at $P < 0.05$.

465

For peer review only

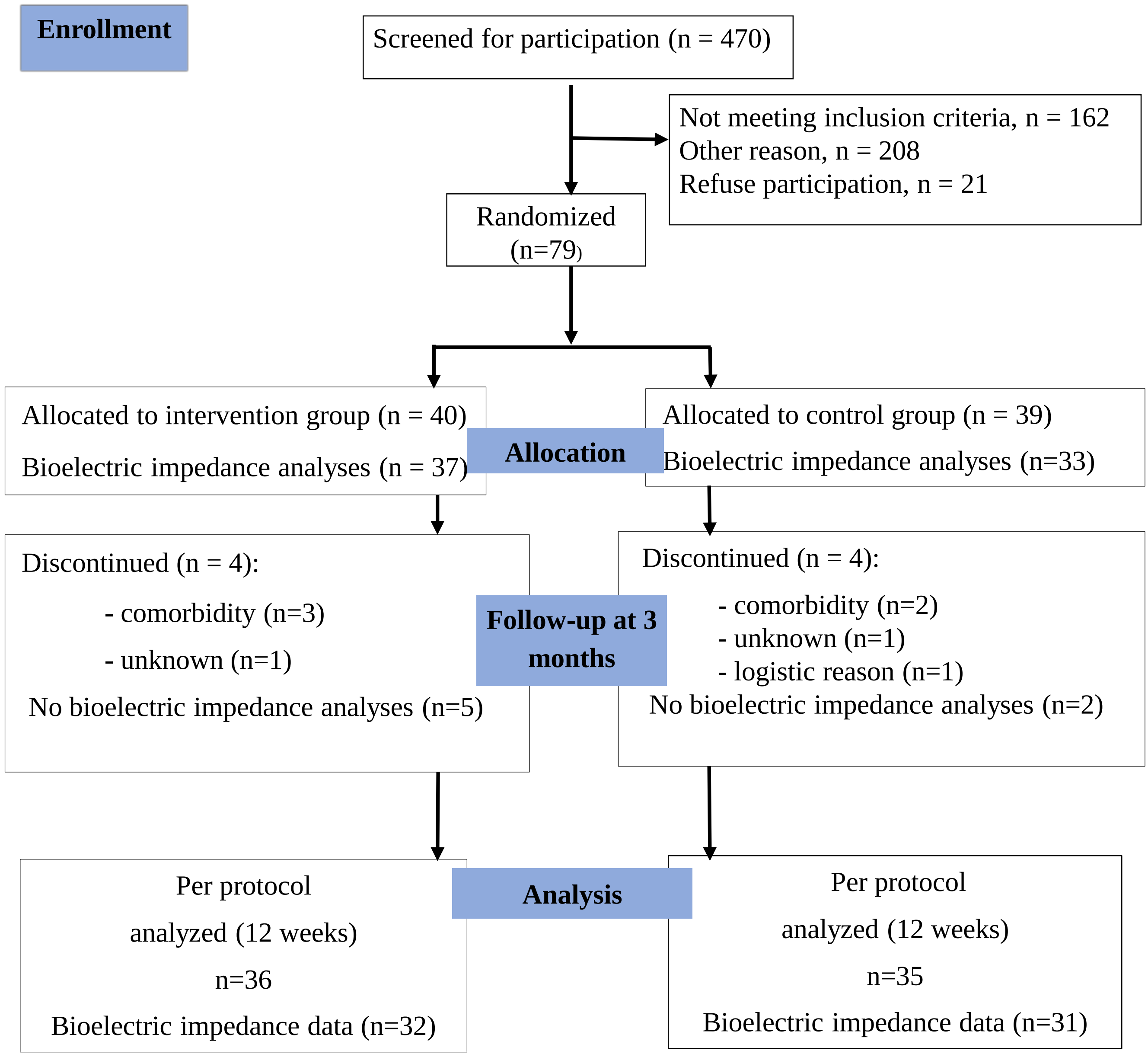
Enrollment
Screened for participation (n = 470)
Not meeting inclusion criteria, n = 162
Other reason, n = 208
Refuse participation, n = 21
Randomized
(n=79)
Allocated to intervention group (n = 40)
Bioelectric impedance analyses (n = 37)
Allocation
Allocated to control group (n = 39)
Bioelectric impedance analyses (n=33)
Discontinued (n = 4):
- comorbidity (n=3)
- unknown (n=1)
No bioelectric impedance analyses (n=5)
Follow-up at 3 months
Discontinued (n = 4):
- comorbidity (n=2)
- unknown (n=1)
- logistic reason (n=1)
No bioelectric impedance analyses (n=2)
Per protocol
analyzed (12 weeks)
n=36
Bioelectric impedance data (n=32)
Analysis
Per protocol
analyzed (12 weeks)
n=35
Bioelectric impedance data (n=31)

# CONSORT 2010 checklist of information to include when reporting a randomised trial*

| Section/Topic | Item No | Checklist item | Reported on page No |
|---|---|---|---|
| **Title and abstract** | | | |
| | 1a | Identification as a randomised trial in the title | Title page |
| | 1b | Structured summary of trial design, methods, results, and conclusions (for specific guidance see CONSORT for abstracts) | Abstract |
| **Introduction** | | | |
| Background and objectives | 2a | Scientific background and explanation of rationale | p.1 |
| | 2b | Specific objectives or hypotheses | p.1-2 |
| **Methods** | | | |
| Trial design | 3a | Description of trial design (such as parallel, factorial) including allocation ratio | p. 2 |
| | 3b | Important changes to methods after trial commencement (such as eligibility criteria), with reasons | N/A |
| Participants | 4a | Eligibility criteria for participants | p. 2 |
| | 4b | Settings and locations where the data were collected | p. 2 |
| Interventions | 5 | The interventions for each group with sufficient details to allow replication, including how and when they were actually administered | p. 5-6 |
| Outcomes | 6a | Completely defined pre-specified primary and secondary outcome measures, including how and when they were assessed | p. 3-5 |
| | 6b | Any changes to trial outcomes after the trial commenced, with reasons | N/A |
| Sample size | 7a | How sample size was determined | p. 6 + previous article (p. 2) |
| | 7b | When applicable, explanation of any interim analyses and stopping guidelines | N/A |
| Randomisation: | | | |
| Sequence generation | 8a | Method used to generate the random allocation sequence | p. 3 |
| | 8b | Type of randomisation; details of any restriction (such as blocking and block size) | p. 3 |
| Allocation concealment mechanism | 9 | Mechanism used to implement the random allocation sequence (such as sequentially numbered containers), describing any steps taken to conceal the sequence until interventions were assigned | p. 3 (previous article , p. 2) |
| Implementation | 10 | Who generated the random allocation sequence, who enrolled participants, and who assigned participants to | p. 3 |

For peer review only


For peer review only - http://bmjopen.bmj.com/site/about/guidelines.xhtml

| | | | |
|---|---|---|---|
| | | interventions | |
| Blinding | 11a | If done, who was blinded after assignment to interventions (for example, participants, care providers, those assessing outcomes) and how | Previous article, p. 2 |
| | 11b | If relevant, description of the similarity of interventions | N/A |
| Statistical methods | 12a | Statistical methods used to compare groups for primary and secondary outcomes | p. 6-7 |
| | 12b | Methods for additional analyses, such as subgroup analyses and adjusted analyses | p. 6-7 |
| **Results** | | | |
| Participant flow (a diagram is strongly recommended) | 13a | For each group, the numbers of participants who were randomly assigned, received intended treatment, and were analysed for the primary outcome | Flow chart |
| | 13b | For each group, losses and exclusions after randomisation, together with reasons | Flow chart |
| Recruitment | 14a | Dates defining the periods of recruitment and follow-up | p. 2 |
| | 14b | Why the trial ended or was stopped | p. 2 |
| Baseline data | 15 | A table showing baseline demographic and clinical characteristics for each group | Table 1 |
| Numbers analysed | 16 | For each group, number of participants (denominator) included in each analysis and whether the analysis was by original assigned groups | Table 1-3 |
| Outcomes and estimation | 17a | For each primary and secondary outcome, results for each group, and the estimated effect size and its precision (such as 95% confidence interval) | Table 3 |
| | 17b | For binary outcomes, presentation of both absolute and relative effect sizes is recommended | N/A |
| Ancillary analyses | 18 | Results of any other analyses performed, including subgroup analyses and adjusted analyses, distinguishing pre-specified from exploratory | N/A |
| Harms | 19 | All important harms or unintended effects in each group (for specific guidance see CONSORT for harms) | p. 9 |
| **Discussion** | | | |
| Limitations | 20 | Trial limitations, addressing sources of potential bias, imprecision, and, if relevant, multiplicity of analyses | p. 11 |
| Generalisability | 21 | Generalisability (external validity, applicability) of the trial findings | Previous article (ref p. 2) |
| Interpretation | 22 | Interpretation consistent with results, balancing benefits and harms, and considering other relevant evidence | p.11 |
| **Other information** | | | |
| Registration | 23 | Registration number and name of trial registry | Title page |
| Protocol | 24 | Where the full trial protocol can be accessed, if available | Title page |
| Funding | 25 | Sources of funding and other support (such as supply of drugs), role of funders | p. 13 |



For peer review only - http://bmjopen.bmj.com/site/about/guidelines.xhtml

*We strongly recommend reading this statement in conjunction with the CONSORT 2010 Explanation and Elaboration for important clarifications on all the items. If relevant, we also recommend reading CONSORT extensions for cluster randomised trials, non-inferiority and equivalence trials, non-pharmacological treatments, herbal interventions, and pragmatic trials. Additional extensions are forthcoming: for those and for up to date references relevant to this checklist, see www.consort-statement.org.

For peer review only


For peer review only - http://bmjopen.bmj.com/site/about/guidelines.xhtml

BMJ Open

# Potential effects on cardio-metabolic risk factors and body composition by short message service (SMS) guided training after recent minor stroke or transient ischemic attack: Post-hoc analyses of the STROKEWALK randomized controlled trial.

| | |
|---|---|
| Journal: | *BMJ Open* |
| Manuscript ID | bmjopen-2021-054851.R1 |
| Article Type: | Original research |
| Date Submitted by the Author: | 21-Sep-2021 |
| Complete List of Authors: | Vahlberg, Birgit; Uppsala Universitet Medicinska fakulteten, Department of Public Health and Caring Sciences, clinical nutrition and metabolism<br>Lundström, Erik; Uppsala Universitet Medicinska fakulteten, Department of Neuroscience<br>Eriksson, Staffan; Centre for Clinical Research, Sörmland, Uppsala University, Eskilstuna<br>Holmback, Ulf; Uppsala Universitet Medicinska fakulteten, Department of Public Health and Caring Sciences, Clinical Nutrition and Metabolism,<br>Cederholm, Tommy; Uppsala Universitet Medicinska fakulteten, Public Health/Clinical Nutrition |
| <b>Primary Subject Heading</b>: | Rehabilitation medicine |
| Secondary Subject Heading: | Neurology, Public health, Nutrition and metabolism |
| Keywords: | Stroke < NEUROLOGY, Neurology < INTERNAL MEDICINE, Rehabilitation medicine < INTERNAL MEDICINE, Stroke medicine < INTERNAL MEDICINE, REHABILITATION MEDICINE |
| | |

SCHOLARONE™
Manuscripts

*I, the Submitting Author has the right to grant and does grant on behalf of all authors of the Work (as defined in the below author licence), an exclusive licence and/or a non-exclusive licence for contributions from authors who are: i) UK Crown employees; ii) where BMJ has agreed a CC-BY licence shall apply, and/or iii) in accordance with the terms applicable for US Federal Government officers or employees acting as part of their official duties; on a worldwide, perpetual, irrevocable, royalty-free basis to BMJ Publishing Group Ltd ("BMJ") its licensees and where the relevant Journal is co-owned by BMJ to the co-owners of the Journal, to publish the Work in this journal and any other BMJ products and to exploit all rights, as set out in our licence.*

*The Submitting Author accepts and understands that any supply made under these terms is made by BMJ to the Submitting Author unless you are acting as an employee on behalf of your employer or a postgraduate student of an affiliated institution which is paying any applicable article publishing charge ("APC") for Open Access articles. Where the Submitting Author wishes to make the Work available on an Open Access basis (and intends to pay the relevant APC), the terms of reuse of such Open Access shall be governed by a Creative Commons licence – details of these licences and which Creative Commons licence will apply to this Work are set out in our licence referred to above.*

*Other than as permitted in any relevant BMJ Author's Self Archiving Policies, I confirm this Work has not been accepted for publication elsewhere, is not being considered for publication elsewhere and does not duplicate material already published. I confirm all authors consent to publication of this Work and authorise the granting of this licence.*

For peer review only

**Potential effects on cardio-metabolic risk factors and body composition by short message service (SMS) guided training after recent minor stroke or transient ischemic attack: Post-hoc analyses of the STROKEWALK randomized controlled trial.**

**Birgit Vahlberg**[1], **Erik Lundström**[2], **Staffan Eriksson**[3,4], **Ulf Holmbäck**[1], **Tommy Cederholm**[1].

[1]Department of Public Health and Caring Sciences, Clinical Nutrition and Metabolism, Uppsala University, Uppsala [2] Department of Neuroscience, Neurology, Uppsala University [3] Centre for Clinical Research, Sörmland, Uppsala University, Eskilstuna [4] Department of Neuroscience, Physiotherapy, Uppsala University.

All authors are from Sweden.

**Corresponding author:** Birgit Vahlberg

Address: Department of Public Health and Caring Sciences, Clinical Nutrition and Metabolism,

Uppsala University, Box 593, BMC

S-751 24 Uppsala, Sweden

Telephone: +46709583473

E-mail: Birgit.Vahlberg@pubcare.uu.se

Word count text: 3085

Word count abstract: 300

Tables: 3

Figures: 1

Clinical trial registration information: URL: http://www.clinicaltrials.gov. Unique identifier (NCT 02902367)

**Keywords:** stroke, transient ischemic attack, exercise, metabolic risk factors, body composition

For peer review only

## Abstract

**Objectives:** To evaluate effects of mobile phone text-messaging exercise instructions on body composition, cardio-metabolic risk markers and self-reported health at three months after stroke.

**Design:** Randomized controlled intervention study with per protocol analyses.

**Setting:** University Hospital in Sweden.

**Participants**: Seventy-nine patients (mean (SD) age 64 (10) years, 37% female) ≥18 years with good motor function (modified Rankin Scale ≤2) and capable to perform six-minute walking test at hospital discharge, were randomized to either intervention (n=40) or control group (n=39). Key exclusion criteria: subarachnoid bleeding, uncontrolled hypertension, severe psychiatric problems or cognitive limitations.

**Interventions:** The intervention group received beyond standard care, daily mobile phone instructional text-messages to perform regular outdoor walking and functional leg exercises. The control group received standard care.

**Main outcome measures:** Fat mass and fat-free mass was estimated by bioelectric impedance analysis. Cardio-metabolic risk factors like blood lipids, glycated haemoglobin (HbA1c) and blood glucose were analyzed at baseline and after three months.

**Results:** Both groups changed favorably in fat-free mass (1.83 kg, 95% CI 0.77 - 2.89; P=0.01, effect size (ES)=0.63 vs 1.22 kg, 95% CI 0.39-2.0; P=0.05, ES=0.54) and fat mass (-1.30 kg, 95% CI -2.45 - -0.14; P=0.029, ES=0.41 vs. -0.76 kg, 95% CI -1.74 - 0.22; P=0.123, ES = 0.28). Also, many cholesterol related biomarkers improved; e.g. total cholesterol -0.65 mmol/L, 95%


For peer review only

CI -1.10 - -0.2; P=0.06, ES: 0.5 vs -1.1 mmol/L, 95% CI -1.47 - -0.56; P>0.001, ES=0.8. However, there were no between group differences. At three months, 94% and 86%, respectively, reported very good/fairly good health in the text messaging and control groups.

**Conclusions:** No clear effect of three months daily mobile phone delivered training instructions was detected on body composition, cardio-vascular biochemical risk factors, or self-perceived health. Further research is needed to evaluate secondary prevention efforts in larger populations after recent stroke.

*Strengths and limitations of this study´*

- This is a randomized controlled clinical trial with an intervention that could be performed anywhere and without extended cost for staff.
- Baseline measurements confirm the potential importance of physical activity showing how sedentary participants had an overall worse metabolic risk-factor profile compared to those with moderate/high physical activity; e.g. higher body mass index, HbA1c, fat-mass and a lower HDL cholesterol at the time for stroke or TIA.
- The paper presents secondary analysis using data from a previous randomized controlled trial.
- The post hoc study was not powered for changes in body composition and cardiovascular risk-markers in blood.

## Introduction

Patients with recent stroke and transient ischemic attacks are at high risk for another vascular event that might lead to permanent disability or death (1). Three out four strokes can be attributed to behavioral risk factors such as smoking, unhealthy diets and sedentary lifestyles. Transient ischemic attack and stroke share the same risk factors (1, 2). A systematic review revealed that people after stroke are highly sedentary, in particular during the acute phase (3), which may affect body composition negatively (1, 4, 5). Loss of muscle mass and increase of fat mass may contribute to insulin resistance and impaired glucose control (4). Beyond increasing muscle mass and reduction of fat mass (5, 6), physical activity and exercise may improve lipid and glucose metabolism.

Physical activity and exercising is of paramount clinical concern, and current recommendations (7) advise people to engage in regular physical exercise of moderate intensity for ≥ 30 min/day (1, 8). The optimal approach to facilitate physical activity for secondary prevention after stroke or transient ischemic attack is not clear (6, 9, 10). The use of mobile-phones in health care are gaining popularity and many apps are available (11). A simple strategy such as Short Message Service (SMS) for optimizing home-based exercising shows potential to be implemented in clinical practice (11). We have recently showed improvements in walking endurance and lower body strength after a three-month mobile-phone guided training program (12).

The importance of body composition and the role of muscle mass in metabolic regulation is recently highlighted (8, 13). Muscle mass is lost and fat mass increased during aging that may result in sarcopenia or sarcopenic obesity (13). A systematic review summarizes the effects of exercise-based interventions on traditional cardio-vascular risk factors, showing improvements of blood pressure, fasting glucose, and fasting insulin, and increasing high-density lipoprotein



For peer review only - http://bmjopen.bmj.com/site/about/guidelines.xhtml
For peer review only

cholesterol in patients after stroke or transient ischemic attack (6). Whether interventions involving self-training after receiving training instructions via SMS could improve cardio-metabolic biochemical risk-factors, or the distribution of body fat and muscles mass has not been studied. Beyond showing improvements in walking endurance and lower body strength, we wanted to analyze this area in more detail (12). In this post-hoc analysis we explored whether effects from the text messaging supported training could induce improvements in body composition and cardio-metabolic risk. Thus the aims of this study in patients after recent stroke or transient ischemic attack were to investigate whether daily mobile phone text messaging coupled with the use of a training diary and a pedometer were better than current standard treatment to improve body composition and cardio-metabolic risk markers and self-reported health at three months after stroke.

## Methods

The STROKEWALK study was a single-center, parallel-group, randomized controlled trial performed at the stroke-unit at the Uppsala University Hospital, Sweden. Ethical approval was obtained from the regional Ethical Review Board of Uppsala University Hospital, Sweden: Dnr: 2015/550, registered with ClinicalTrials.gov (NCT02902367). We used the CONSORT checklist when writing our report (14)

Design, recruitment, determination of sample size, inclusion criteria and intervention have been described in detail elsewhere (12). In summary, inclusion criteria were age 18 years or older with minor stroke (infarction or intracerebral hemorrhage; first-ever or recurrent) or transient ischemic attack; with cognition and motor capacity, e.g, walking endurance, enough to perform the 6-minute walking test (with or without walking aid).

For peer review only



For peer review only - http://bmjopen.bmj.com/site/about/guidelines.xhtml

*Procedure*

Recruitment started November 2016, and the last follow-up assessment was performed December 2018. Patients were evaluated by one physiotherapist (BV), and a research assistant performed the body composition measurements at median five days after verified event. All participants gave oral and written informed consent to be included in the study.

Study participants underwent a physical examination, laboratory tests and answered questionnaires. Demographic and comorbidity data were collected from the medical records. Blood pressure was registered as the last measurement before leaving hospital. We calculated the Charlson Comorbidity Index – an instrument that predicts 10-year survival (15) – to classify comorbidity. Information on self-reported health after three months and one year were obtained from the Swedish quality Riksstroke registry. Education level was divided into university studies or not. Smokers were grouped into daily-smokers and non-smokers. Ex-smokers were categorized as non-smokers.

*Randomization*

The allocation to either intervention or control group was based on randomization, 1:1 ratio, stratified by gender. The random allocation sequence is described more thoroughly in the primary outcome paper (12).

*Baseline examinations*

Examinations at baseline have been extensively described previously (10). In summary we used

- The Montreal Cognitive Assessment scale (0-30 points) to evaluate cognitive function (16).
- The modified Rankin Scale (range 0 [no symptoms] to 6 [death]) (17).



For peer review only - http://bmjopen.bmj.com/site/about/guidelines.xhtml

- The 6-minute walking test (meters) as a sub-maximal exercise test (18). Participants walked for 6 minutes over a 30 m course.
- Stress profile: The questionnaire refers to stress behaviors in everyday life and the stress profile was assessed by a four-level self-reported scale. The scale combines 20 claims with agreements expressed in terms of time urgency/impatience or easily aroused irritation/hostility; for example, other people ´s mistake irritate me (19). It is scored from not at all to fully agree, 0 to 3.
- The 4-level Saltin Grimby Physical Activity Level Scale was used to measure self-reported leisure-time physical activity during the past year before stroke (20).
- The European Working group on Sarcopenia in Older People-2 definition for sarcopenia; i.e. probable sarcopenia is indicated by low muscle strength by the five-times chair-stand test > 15 seconds or handgrip strength (Jamar® dynamometer) <27 kg in men and <16 kg in women, respectively (13). Sarcopenia was confirmed by fat-free mass index (see below) < 17 $kg/m^2$ for men and 15 $kg/m^2$ for women.

*Outcome assessments for post-hoc analyses*

The study included several endpoints at three months, i.e., body composition changes in fat-free mass and fat mass by bioelectric impedance analyses, and biochemical cardio-metabolic risk factors, and nutritional status.

The fat-free mass (kg) and fat-mass (kg) were measured with the bioelectric impedance analyses device Inbody S20; Inbody, Seoul, South Korea. Measurements were performed in the supine position. Fat-free mass index and fat-mass index was calculated by dividing masses in kg by height (m) squared.



For peer review only - http://bmjopen.bmj.com/site/about/guidelines.xhtml

Body mass index (BMI) was calculated as body weight (kg) divided by height (m) squared. The participants were divided into categories: underweight (< 18.5 kg/ $m^2$), normal weight (18.5 to 24.9 kg/ $m^2$), overweight (25-29.9 kg/ $m^2$), obese class I (30-35 kg/ $m^2$), class II (>35 – 39.9 kg/ $m^2$) and class III (≥40 kg/ $m^2$) (1).

Non-fasting venous blood samples for baseline analyses of glycated hemoglobin (HbA1c), serum insulin-like growth factor and biochemical cardio-metabolic risk factors were obtained. The samples were frozen at -70 ° until analyses. The biochemical analyses were performed by accredited methods of the Clinical Chemistry Laboratory at the Uppsala University Hospital, Uppsala.

The cardio-metabolic biochemical risk factors total, LDL and HDL cholesterol, and triglycerides were analyzed. Baseline values of blood lipids and C-reactive protein were recorded from values obtained at admission to the emergency ward or the day after admission to the stroke-unit. A C-reactive protein level of >5 mg/l was considered to indicate ongoing inflammation.

Non-fasting blood samples at three months follow-up were collected and frozen at –70° C until analyses. The serum lipid concentrations were considered pathological when the non-HDL cholesterol (total cholesterol minus HDL cholesterol) was >2.2 mmol/L; LDL cholesterol >1.4 mmol/L and high-density lipoprotein cholesterol was ≤1.2 mmol/L in women and ≤1.1 mmol/L in men (8), respectively.

Self-reported health at three months and twelve months were analyzed from data retrieved from the Riksstroke national registry. Reported answers were very good, fairly good, fairly bad, very bad health. Data were only available for the patients with stroke, not for patients with TIA.



For peer review only - http://bmjopen.bmj.com/site/about/guidelines.xhtml

Mortality at one year was checked by reviewing the individual's medical records that is connected to the official death records.

*Text messaging intervention*

As previously described, the intervention was an add-on to standard care and comprised three different strategies: 1) daily text-messaging (short message service) with instructions what and how to exercise, 2) training diaries for three months and 3) pedometers for step counts registration at the first and last week of the study (12).

The daily morning text messages across the study period gave instructions on how to exercise to increase walking endurance and lower body strength. There was no possibility to text back for help or advice. The training program was initiated about one week after the patients had been randomized.

*Control intervention*

Patients in the control group were given standard post-stroke care, which usually does not include specific advice about physical activity or home exercising. The control participants had no restrictions regarding physical activity, exercise or taking part in rehabilitation services.

*Statistical analyses*

No a priori power analysis was performed in the present post-hoc study and statistical calculations were based on per protocol analysis. Data and variables were checked for missing values and potential outliers.



For peer review only - http://bmjopen.bmj.com/site/about/guidelines.xhtml

Categorical data are presented as percentage and descriptive data as mean and SD or median and IQR. To check for normal distribution, the Shapiro-Wilk W test and histogram viewing were used.

Significance of between group differences in physical activity at baseline were analyzed by Pearson´s chi-squared test ($\chi^2$) with Fishers exact test for nominal data or the Kruskal-Wallis test for non-normal or ordinal data. One-way ANOVA tests with Bonferroni corrections were used to analyze continuous normal-distributed data.

Differences in changes between baseline and the three-month follow-up between the text messaging group and the control group are presented with 95% CI and tested by non-parametric methods (Mann-Whitney U test) for ordinal or non-normally distributed variables. For all other data, parametric methods (the independent student´s t-test) were used.

Within group differences; i.e., the difference between baseline and the three-month follow-up were tested using the paired samples t-test or the Wilcoxon's signed rank test. Correlation strength was calculated by Spearman rho coefficient.

The statistical Package for the Social Sciences (SPSS), version 27, was used for the analyses (SPSS Inc. Chicago, IL, USA).The level of statistical significance was set at $P < 0.05$.

## Results

*Baseline characteristics*

Figure 1 summarizes the recruitment and participant flow in a CONSORT diagram. Out of 100 individuals that were eligible for the study, 79 were invited to participate. A total of 71 individuals completed the full study period; i.e., a retention rate of 90%. Table 1 gives bbaseline characteristics of the text messaging- and control groups. Fifteen percent of the included



For peer review only - http://bmjopen.bmj.com/site/about/guidelines.xhtml

individuals had diabetes mellitus type 2. At baseline, a total of eleven individuals (15%) had a C-reactive protein level > 5 mg/L. At baseline, individuals with HbA1C ≥ 42 mmol/mol were significantly more sedentary compared to those with moderate to high physical activity, as measured with the Saltin Grimby Physical Activity Level Scale.

Table 2 displays baseline characteristics with subjects divided into groups according to the Saltin-Grimby Physical Activity Level Scale at baseline. Sedentary participants compared to those with moderate/high physical activity had an overall worse risk-factor profile with significant differences; e.g. higher body mass index, HbA1c, fat-mass and a lower HDL cholesterol. Missing data are reported.

The body mass index range was 20.1 $kg/m^2$ to 42.8 $kg/m^2$. At baseline, fifteen percent had probable sarcopenia according to either a reduced chair-stand test or reduced handgrip strength. None had confirmed sarcopenia, i.e. reduced lower body strength combined with low muscle mass.

*Changes of body composition and cardio-metabolic risk markers during the intervention period*

Table 3 shows that on average there was no weight change in neither the text messaging group nor in the control group at three months, whereas variability in weight changes in both groups were substantial; i.e., from –10.1 to 7.8 kg.

A positive within group effect on HDL cholesterol was seen in female participants that received daily text messages, but there were no between group effects.

Fat-free mass increased significantly in both groups, with no difference between groups (Table 3). Concurrently, fat-mass decreased in both groups, but again no difference between groups was observed.



For peer review only - http://bmjopen.bmj.com/site/about/guidelines.xhtml

Table 3 shows that total cholesterol, LDL and HDL cholesterol changed in beneficial directions over the three-month period irrespective of group allocation, but no significant difference between groups were found.

Only minor changes were observed for HbA1c in both groups. Serum insulin-like growth factor tended to decrease in both groups, but no significant difference between groups were found. Although only 37% of the participants were women, we performed sex-specific subgroup analyses of body composition variables as well as of biochemical cardio-metabolic risk-markers, which did not change the results.

At baseline, C-reactive protein was not associated with any of the cardiovascular risk-markers nor fat-free mass, fat mass or self-reported physical activity level.

*Mortality, self-reported health and adverse events*

One participant of the control group had passed away at one year. Data on self-reported health at three months and one year after the event could be retrieved in 62 and 50 of all participating individuals with stroke. No difference between the groups was registered; i.e., at three months 94% and 86%, respectively, reported very good or fairly good health in the text messaging and control groups. At one year 89% and 86%, respectively, reported very good or fairly good health in the text messaging and control groups.

No adverse events such as fall-related fractures, syncope or dizziness events requiring hospitalization until three months follow-up were reported.

*Patient and public involvement*



For peer review only - http://bmjopen.bmj.com/site/about/guidelines.xhtml

Patients were involved in the decision on how to deliver the training instructions; i.e., by text messages or video-links by SMS. In a pilot study (21) we investigated whether patients after stroke preferred text-SMS or SMS with video (You-Tube) instructions, with a preference for the first approach. Otherwise, there was no direct patient or public involvement.

## Discussion

This study is a post hoc analysis of the STROKEWALK study, where a text messaging intervention was previously reported to increase the walking capacity as well as chair-stand capacity, i.e. the primary outcomes of the study (22). However, the post hoc analyses showed that beneficial effects on body composition and some biochemical cardio-metabolic risk factors were achieved in both groups, irrespective if they received text messages or not. Thus, the positive effects on walking capacity in the intervention group did not transform to beneficial effects on body composition or the selected group of biochemical cardio-metabolic risk factors in the intervention group only.

It is well-known that weight reduction and regular physical exercise improve insulin sensitivity and decreases triglyceride levels (8). Aerobic physical activity such as 25-30 km brisk walking per week may increase HDL cholesterol by 0.08-0.15 mmol/L (8). Overweight and especially abdominal adiposity contributes to overall dyslipidemia and these conditions were common in this study population that also showed both excessive weight loss and weight gain after three months. Unfortunately, we were not able to control for fluctuation in weight or depressive symptoms that became present during the study period. Both the text messaging group and the control group increased their fat-free mass, whereas fat-mass decreased only in the text messaging intervention group. A similar positive within group effect on HDL cholesterol was



For peer review only - http://bmjopen.bmj.com/site/about/guidelines.xhtml

observed in female participants that received daily text messages, which is in line with the positive effects on walking and lower body strength after the training intervention. A Cochrane review concluded that there is currently limited high quality research to support lifestyle interventions targeting mortality, blood pressure, lipid profiles and insulin resistance after stroke or transient ischemic attack (23). Treatment targets for extended secondary prevention (8) for body weight could be body mass index <25 kg/m$^2$, moderately vigorous physical activity for 30-60 minutes most days, and for individuals with diabetes HbA1c <53 mmol/mol.

In recent years the importance of muscle mass for mobility, muscle strength, energy, lipid and glucose metabolism has been highlighted in rehabilitation (13, 24). Probable sarcopenia, i.e. low muscle strength and confirmed sarcopenia, i.e. the addition of low muscle mass is gaining increasing interest as risk factors for falls and fractures. Impaired activities of daily living are associated with cardiac and respiratory disease, as well as to cognitive impairment (13). At baseline, twelve participants displayed probable sarcopenia. Thus, interventions to improve muscle function are important in a population that have suffered from stroke or transient ischemic attack. Many already have a low level of physical activity before the cerebrovascular event, and after the event the activity level might become even lower if no actions are taken.

For some individuals the walking program and the number of repetitions of chair-rising might have been too low to increase muscle mass. For this purpose individualized exercise instructions with higher intensity might be needed. There are not always opportunities for supervised exercise for those with minor stroke or transient ischemic attack when discharged from the hospital. Therefore methods including tele-rehabilitation and self-training can be an alternative or complement to usual rehabilitation, especially in remote or underserved areas (25).



For peer review only

For peer review only - http://bmjopen.bmj.com/site/about/guidelines.xhtml

As this was an explorative post-hoc study, power was not calculated for the outcomes in this study. It is obvious that this post-hoc study might have been underpowered to show any differences in body composition or cardio-metabolic risk factors. The study population of the current study showed great variability in many measures, which may have implications for the necessary sample size. The effects of exercise are known to be strongest in individuals that are sedentary (26). Approximately 14% of the participants in this study were sedentary at study start. By chance nine of the eleven sedentary individuals were randomized to the control group, which might have had an impact on the results. Other potential reasons for the weak effects could be that the time period was too short to induce stronger effects, or the substantial fluctuations in weight.

In conclusion, the results of this study should be interpreted with caution, since they are the outcome of secondary analyses of a previously reported randomized controlled trial. We observed that body composition; i.e. fat-free mass and fat mass, as well as some cardio-metabolic risk factors like various cholesterol fractions, improved slightly three months after a stroke or transient ischemic attack both in the text messaging group and in the control group. A majority of the participants reported good or fairly good health after three months. The study indicates that not a negligible amount of individuals after stroke have cardiovascular risk-factors that need to be considered for in secondary prevention.

*Clinical messages*

- Body composition, HDL and LDL cholesterol improved in post-stroke patients after three months irrespective of the intervention.



For peer review only - http://bmjopen.bmj.com/site/about/guidelines.xhtml

- Additional studies are needed in larger study populations over longer study periods to elucidate if potential cardio-metabolic beneficial effects could be achieved.
- Especially sedentary individuals need help how to increase physical activity and reduce risk of recurrent cerebrovascular events.

**Figure 1.** CONSORT diagraming. Flow chart of the study participants through the phases of the original randomized controlled trial. Data are collected in Uppsala University Hospital, Sweden (November 2016 - December 2018).

**Conflicts of interest:** None.

**Author contributions:** This study was conceived, organized and managed by BV, EL, UH, SE and TC. All authors listed above contributed to the study design and data interpretation. Writing of the first draft of the paper was done by BV and all authors were involved in preparation and critique of the manuscript. The Uppsala Research Center assisted with some statistical analyses.

**Funding:** This study was funded by the Medical Faculty at Uppsala University, the Swedish Stroke Association (Stroke-Riksförbundet), the Geriatric funding and the Swedish Associations of Physiotherapists, Neurology.

**Acknowledgement**

We thank research assistant Mia Berglund for the co-operation in the execution of the study. We also thank Marina Lahti (www.intime.se) for assisting with sending the text messages.

**Data availability statement**



For peer review only - http://bmjopen.bmj.com/site/about/guidelines.xhtml

All data relevant to the study are included in the article or uploaded as supplementary information.

**Ethics statement**

Patients consent for publication: Obtained.

For peer review only



For peer review only - http://bmjopen.bmj.com/site/about/guidelines.xhtml

## REFERENCES


1. Virani SS, Alonso A, Benjamin EJ, Bittencourt MS, Callaway CW, Carson AP, et al. Heart Disease and Stroke Statistics-2020 Update: A Report From the American Heart Association. Circulation. 2020;141(9):e139-e596.
2. RIKSstroke. RIKSstroke, the Swedish Stroke Register. availble from:htpp://www.riksstroke.org; (cited January, 2021); .
3. Fini NA, Holland AE, Keating J, Simek J, Bernhardt J. How Physically Active Are People Following Stroke? Systematic Review and Quantitative Synthesis. Phys Ther. 2017;97(7):707-17.
4. English C, McLennan H, Thoirs K, Coates A, Bernhardt J. Loss of skeletal muscle mass after stroke: a systematic review. Int J Stroke. 2010;5(5):395-402.
5. English C, Thoirs K, Coates A, Ryan A, Bernhardt J. Changes in fat mass in stroke survivors: a systematic review. Int J Stroke. 2012;7(6):491-8.
6. D'Isabella NT, Shkredova DA, Richardson JA, Tang A. Effects of exercise on cardiovascular risk factors following stroke or transient ischemic attack: a systematic review and meta-analysis. Clin Rehabil. 2017;31(12):1561-72.
7. World, Health, Organization. World Health Organization. WHO guidelines on physical activity and sedentary behaviour 2020. Geneva, Switzerland: World Health Organization, 2020. https://www.who.int/publications/i/item/9789240015128 (23 April 2021).
8. Mach F, Baigent C, Catapano AL, Koskinas KC, Casula M, Badimon L, et al. 2019 ESC/EAS Guidelines for the management of dyslipidaemias: lipid modification to reduce cardiovascular risk. Eur Heart J. 2020;41(1):111-88.
9. Heron N, Kee F, Cardwell C, Tully MA, Donnelly M, Cupples ME. Secondary prevention lifestyle interventions initiated within 90 days after TIA or 'minor' stroke: a systematic review and meta-analysis of rehabilitation programmes. Br J Gen Pract. 2017;67(654):e57-e66.
10. Moore SA, Hallsworth K, Plötz T, Ford GA, Rochester L, Trenell MI. Physical activity, sedentary behaviour and metabolic control following stroke: a cross-sectional and longitudinal study. PLoS One. 2013;8(1):e55263.
11. Gandhi S, Chen S, Hong L, Sun K, Gong E, Li C, et al. Effect of Mobile Health Interventions on the Secondary Prevention of Cardiovascular Disease: Systematic Review and Meta-analysis. Can J Cardiol. 2017;33(2):219-31.
12. Vahlberg B, Lundström E, Eriksson S, Holmbäck U, Cederholm T. Effects on walking performance and lower body strength by short message service guided training after stroke or transient ischemic attack (The STROKEWALK Study): a randomized controlled trial. Clin Rehabil. 2021;35(2):276-87.
13. Cruz-Jentoft AJ, Bahat G, Bauer J, Boirie Y, Bruyère O, Cederholm T, et al. Sarcopenia: revised European consensus on definition and diagnosis. Age Ageing. 2019;48(4):601.
14. Schulz KF, Altman DG, Moher D, Group C. CONSORT 2010 Statement: Updated guidelines for reporting parallel group randomised trials. J Clin Epidemiol. 2010;63(8):834-40.
15. Goldstein LB, Samsa GP, Matchar DB, Horner RD. Charlson Index comorbidity adjustment for ischemic stroke outcome studies. Stroke. 2004;35(8):1941-5.
16. Nasreddine ZS, Phillips N, Chertkow H. Normative data for the Montreal Cognitive Assessment (MoCA) in a population-based sample. Neurology. 2012;78(10):765-6; author reply 6.




For peer review only - http://bmjopen.bmj.com/site/about/guidelines.xhtml

17. Banks JL, Marotta CA. Outcomes validity and reliability of the modified Rankin scale: implications for stroke clinical trials: a literature review and synthesis. Stroke. 2007;38(3):1091-6.
18. ATS statement: guidelines for the six-minute walk test. Am J Respir Crit Care Med. 2002;166(1):111-7.
19. Claesson M, Birgander LS, Lindahl B, Nasic S, Aström M, Asplund K, et al. Women's hearts--stress management for women with ischemic heart disease: explanatory analyses of a randomized controlled trial. J Cardiopulm Rehabil. 2005;25(2):93-102.
20. Rödjer L, Jonsdottir IH, Rosengren A, Björck L, Grimby G, Thelle DS, et al. Self-reported leisure time physical activity: a useful assessment tool in everyday health care. BMC Public Health. 2012;12:693.
21. Vahlberg B, Holmbäck U, Eriksson S, Cederholm T. Protocol and pilot study of a short message service-guided training after acute stroke/transient ischemic attack to increase walking capacity and physical activity. Prev Med Rep. 2018;11:109-14.
22. Vahlberg B LE, Eriksson S, Holmbäck U, Cederholm T. Effects on walking performance and lower body strength by short message service guided training after stroke or transient ischemic attack (The STROKEWALK Study): a randomized controlled trialb>. Clinical Rehabilitation. 2020, September 18;Online.
23. Mackay-Lyons M, Thornton M, Ruggles T, Che M. Non-pharmacological interventions for preventing secondary vascular events after stroke or transient ischemic attack. Cochrane Database Syst Rev. 2013;3:CD008656.
24. Biolo G, Cederholm T, Muscaritoli M. Muscle contractile and metabolic dysfunction is a common feature of sarcopenia of aging and chronic diseases: From sarcopenic obesity to cachexia. Clin Nutr. 2014.
25. Tchero H, Tabue Teguo M, Lannuzel A, Rusch E. Telerehabilitation for Stroke Survivors: Systematic Review and Meta-Analysis. J Med Internet Res. 2018;20(10):e10867.
26. Virani SS, Alonso A, Aparicio HJ, Benjamin EJ, Bittencourt MS, Callaway CW, et al. Heart Disease and Stroke Statistics-2021 Update: A Report From the American Heart Association. Circulation. 2021;143(8):e254-e743.

For peer review only



For peer review only - http://bmjopen.bmj.com/site/about/guidelines.xhtml

Table.1 Baseline characteristics of individuals allocated to the text messaging or control group. Data given as n (%) if not otherwise stated.

| | **Text messaging (N=40)** | **Control group (N=39)** |
|---|---|---|
| Sex, male | 26 (67) | 24 (60) |
| Age (years), mean (SD) | 63.9 (10) | 63.9 (10) |
| **Type of stroke,** | | |
| Cerebral infarction | 29 (72) | 28 (72) |
| Intra-cerebral hemorrhage | 4 (10) | 5 (13) |
| Transient Ischemic Attack | 7 (18) | 6 (15) |
| **Lifestyle factors** | | |
| Saltin Grimby Physical Activity Level Scale, score | | |
| Sedentary | 2 (5) | 9 (23) |
| Light physical activity | 29 (73) | 24 (62) |
| Moderate/high physical activity | 9 (22) | 6 (15) |
| Body Mass Index (kg/$m^2$ ), mean SD | 27.3 (4.2) | 27.7 (4.9) |
| Normal weight <25 | 12 (30.0) | 12 (30.8) |
| Overweight, 25-29.9 | 17(42.5) | 17 (43.6) |
| Obese, class I 30-35 | 9 (22.5) | 6 (15.4) |
| Obese, class II >35–39.9 | 2 (5.0) | 3 (7.7) |
| Obese, class III ≥40 | 0 | 1 (2.6) |
| Stress profile, score, | | |
| <25 (not stressed) | 27 (68) | 25(64) |
| 25-34 (sometimes stressed) | 11(27) | 7(18) |



For peer review only - http://bmjopen.bmj.com/site/about/guidelines.xhtml

| | | |
|---|---|---|
| ≥35 (highly stressed) | 2 (5) | 7 (18) |
| Diabetes mellitus type 2 | 5 (12) | 7 (18) |
| Glycated hemoglobin (HbA1C), mmol/mol, mean(SD) | 37.6 (7.6) | 38.4 (7.6) |
| Non diabetic | 36.4 (4.2) | 35.3 (3.4) |
| Systolic blood pressure, mean(SD) | 129.1 (14.5) | 132.2 (18.5) |
| >140 | 4 (10) | 11 (30) |
| Diastolic blood pressure, mean(SD) | 75.1 (11.4) | 77.0 (10.7) |
| >90 | 2 (5) | 3 (8) |

For peer review only



For peer review only - http://bmjopen.bmj.com/site/about/guidelines.xhtml

Table 2. Level of physical activity at baseline, reported from the Saltin Grimby Physical Activity Scale.

| | Missing values n (%) | All | Sedentary | Light physical activity | Moderate/high physical activity | P-value |
|---|---|---|---|---|---|---|
| n (%) | | 79 (100) | 11 (14) | 53 (68) | 15 (19) | |
| Women, n (%) | 0 | 29 (37) | 2 (18) | 24 (45) | 3 (20) | |
| Age, yrs , mean (SD) | 0 | 63.9 (10.4) | 61.8 (9.0) | 64.0 (11.3) | 64.9 (8.0) | 0.75 |
| Diabetes mellitus, yes n (%) | 0 | 12 (15) | 3 (27) | 8 (15) | 1 (7) | 0.45 |
| University studies, yes n (%) | 0 | 40 (62) | 5 (46) | 26 (49) | 9 (60) | 0.79 |
| Non-smoker, n (%) | 0 | 71 (90) | 8 (73) | 48 (91) | 15 (100) | 0.087 |
| High intake fruit and vegetables, n (%) | 2 (2.5 %) | 55 (70) | 6 (54) | 36 (69) | 13 (93) | 0.08 |
| Body weight, kg, median (IQR) | 0 | 80.90 (25.1) | 97.80 (28.60) | 80.90 (22.90) | 77.3 (11.40) | **0.027** |
| Body Mass Index, $kg/m^2$, median (IQR) | 0 | 26.90 (5.70) | 30.90 (6.50) | 27.0 (5.15) | 24.90 (2.4) | **0.016** |
| Fat-free mass, kg, mean (SD) | 9 (11.4 %) | 57.61 (11.10) | 64.18 (10.86) | 55.72 (11.31) | 58.92 (9.45) | 0.08 |
| Fat-free mass index, $kg/m^2$, mean (SD) | 9 (11.4 %) | 18.91 (2.14) | 20.0 (2.24) | 18.75 (2.16) | 18.64 (1.92) | 0.20 |
| Fat-free mass percent, mean (SD) | 9 (11.4 %) | 70 (10) | 68 (10) | 69 (9.8) | 74 (9.9) | 0.21 |
| Fat-mass, kg, mean (SD) | 9 (11.4 %) | 25.10 (10.9) | 32.0 (13.0) | 24.92 (10.35) | 18.96 (8.47) | **0.042** |
| Fat- mass index, $kg/m^2$, mean (SD) | 9 (11.4 %) | 8.40 (3.69) | 10.0 (4.13) | 8.58 (3.63) | 6.79 (3.13) | 0.087 |
| Fat- mass percent, mean (SD) | 9 (11.4%) | 30 (10) | 32 (10) | 30 (9.8) | 26 (9.8) | 0.20 |
| Systolic blood pressure, (mm Hg), mean (SD) | 4 (5 %) | 131 (16.6) | 137 (18.9) | 129 (15.6) | 132 (17.7) | 0.29 |
| Diastolic blood pressure, (mm Hg), median(IQR) | 4 (5 %) | 75 (15) | 80 (10) | 70 (10) | 85 (20) | 0.13 |

For peer review only

| | | | | | | |
|---|---|---|---|---|---|---|
| P - Cholesterol, (mmol/l), mean (SD) | 3 (3.8 %) | 5.13 (1.20) | 4.52 (1.17) | 5.20 (1.19) | 5.33 (1.19) | 0.18 |
| P – LDL cholesterol, (mmol/l), median (IQR) | 3 (3.8 %) | 3.20 (1.78) | 2.60 (1.50) | 3.20 (1.80) | 3.60 (2.13) | 0.61 |
| P – HDL cholesterol, (mmol/l), median (IQR) | 3 (3.8 %) | 1.30 (0.50) | 0.99 (0.37) | 1.30 (0.60) | 1.35 (0.42) | **0.018** |
| Women, mean (SD), | | 1.48 (0.42) | 1.11 (0.27) | 1.51 (0.44) | 1.55 (0.07) | |
| Men, median (IQR) | | 1.20 (0.43) | 0.99 (0.33) | 1.20 (0.43) | 1.25 (0.45) | |
| P – LDL/HDL ratio, (mmol/L), mean (SD) | 3 (3.8 %) | 2.57 (1.05) | 2.86 (1.11) | 2.46 (1.04) | 2.71 (1.07) | 0.45 |
| P - Triglycerides, (mmol/l), median (IQR) | 3 (3.8 %) | 1.22 (0.62) | 1.49 (0.45) | 1.23 (0.64) | 1.11 (0.91) | 0.70 |
| P - Glucose, (mmol/l), median (IQR) | 13(16.4 %) | 6.90 (1.70) | 7.60 (2.0) | 6.65 (1.60) | 6.90 (1.70) | 0.55 |
| P - HbA1c, (mmol/mol), median (IQR) | 3 (3.8 %) | 36.0 (5.0) | 39.0 (26.0) | 35.0 (6.0) | 35.0 (5.0) | 0.053 |
| P - C-reactive protein, (mg/L), median (IQR) | 6 (7.6%) | 1.70 (2.3) | 1.40 (1.63) | 2.00 (2.44) | 1.10 (3.15) | 0.51 |
| S - Insulin-like growth factor 1 (μ/L), mean (SD) | 13 (16.4 %) | 161.8 (41.0) | 189.9 (57.0) | 156.7 (36.4) | 160.8 (40.2) | 0.11 |

Significance of between group differences was analyzed by Pearsons chi-squared test with Fishers exact test for nominal data or the Kruskal-Wallis test for non-normal or ordinal data and the one-way ANOVA test with Bonferroni correction for continuous normal distributed data. The significance level was set at $P < 0.05$.

For peer review only

For peer review only - http://bmjopen.bmj.com/site/about/guidelines.xhtml

Table 3. Baseline and follow-up outcome of cardio-metabolic risk factors and body composition by group, analyzed as per protocol. Data are presented as means (SD) or median (IQR).

| | | Text messa-ging group | | | | Control group | | | | |
|---|---|---|---|---|---|---|---|---|---|---|
| | n | Baseline | 3 month | *P* | n | Baseline | 3 month | *P* | Mean/median difference (95%CI) | P-value |
| Women | 16 | | | | 13 | | | | | |
| Body weight, kg, median(IQR) | 33 | 81.9 (15.2) | 82.4 (15.2) | 0.28 | 31 | 83.3(30.1) | 83.7 (17.7) | 0.43 | -0.2(-1.40 to 0.8) | 0.45 |
| Body Mass Index, kg/m$^2$ median(IQR) | 33 | 26.6 (6.0) | 26.36 (6.0) | 0.69 | 31 | 27.30 (5.9) | 26.36 (6.0) | 0.98 | -0.026(-0.47 to 0.36) | 0.36 |
| Fat-free mass, kg mean(SD) | 32 | 57.2 (9.4) | 59.0 (10.3) | **0.001** | 31 | 58.65 (12.9) | 59.86 (13.1) | **0.005** | -0.62(-1.9 to 0.70) | 0.35 |
| Fat-free mass%, mean(SD) | 32 | 71 (10) | 72 (10) | **0.014** | 31 | 71(10) | 72(10) | **0.029** | -0.006(-0.21 to 0.009) | 0.43 |
| Fat-free mass index, kg/m$^2$, mean(SD) | 32 | 18.93 (1.9) | 19.52 (2.0) | **0.001** | 31 | 19.00 (2.4) | 19.41 (2.5) | **0.004** | -0.18(-0.6 to 0.24) | 0.39 |
| Fat mass, kg, mean(SD) | 32 | 24.69 (11.5) | 23.39 (11.2) | **0.029** | 31 | 24.65 (10.9) | 23.89 (10.0) | 0.123 | 0.74(-0.95 to 2.0) | 0.48 |
| Fat mass%, mean(SD) | 32 | 29.3 (10) | 27.6 (10) | **0.012** | 31 | 29.1 (10) | 28.2 (9.6) | **0.041** | 0.007(-0.008 to 0.022) | 0.33 |

For peer review only

| | | | | | | | | | | |
|---|---|---|---|---|---|---|---|---|---|---|
| Fat mass index, kg/m$^2$, mean (SD) | 32 | 8.30 (3.9) | 7.89 (3.8) | **0.036** | 31 | 8.19 (3.7) | 7.93 (3.4) | 0.103 | -0.16(-0.64 to 0.33) | 0.52 |
| P-Cholesterol,(mmol/l) | 35 | 5.13 (1.19) | 4.47 (1.0) | **0.006** | 32 | 5.11 (1.23) | 4.09 (1.15) | **<0.001** | -0.36(-0.99 to 0.26) | 0.25 |
| P-non- HDL cholesterol (mmol/L), mean(SD) | 34 | 3.83 (1.16) | 2.95 (1.00) | **<0.001** | 32 | 3.83 (1.0) | 2.65 (0.95) | **<0.001** | -0.29(-0.89 to 0.31) | 0.34 |
| P-LDL cholesterol, (mmol/l), mean(SD) | 34 | 3.17 (1.15) | 2.37 (0.88) | **<0.001** | 33 | 3.13 (1.0) | 2.37 (0.80) | **<0.001** | -0.30(-0.83 to 0.24) | 0.28 |
| P-HDL cholesterol. (mmol/l), mean(SD) | 34 | 1.32 (0.33) | 1.53 (0.41) | **<0.001** | 33 | 1.30 (0.48) | 1.45 (0.50) | **<0.001** | -0.03 (-0.13 to 0.07) | 0.55 |
| Women | 13 | 1.42 (0.37) | 1.69 (0.44) | **0.001** | 10 | 1.56 (0.57) | 1.61 (0.58) | 0.24 | -0.16 (-0.35 to 0.03) | 0.10 |
| Men | 21 | 1.26 (0.30) | 1.43 (0.37) | **0.003** | 23 | 1.18 (0.40) | 1.38 (0.50) | **<0.001** | 0.037(-0.08 to 0.15) | 0.54 |
| P-LDL/HDL cholesterol, ratio, mean(SD) | 34 | 2.54 (1.00) | 1.66 (0.72) | **<0.001** | 32 | 2.64 (1.05) | 1.53 (0.74) | **<0.001** | -0.23 (-0.67 to 0.21) | 0.29 |
| Glycated hemoglobin, (mmol/mol), median (IQR) | 35 | 35.5 (5.0) | 37.0 (5.8) | 0.067 | 32 | 36.0 (5.0) | 37.0 (5.0) | 0.11 | 0(-1 to 1) | 0.81 |
| S-Insulin-like growth factor-1 (µ/L), mean (SD) | 29 | 167.9 (45.2) | 152.8 (52.8) | 0.061 | 33 | 157.6 (37.6) | 149.6 (41.4) | 0.086 | 7.0(-10.36 to 24.44) | 0.42 |

For peer review only

All within group values are presented as mean (SD). Significance of between group differences in glycated hemoglobin, weight and body mass index was analyzed by the Mann-Whitney U test. The median estimate was calculated using the Hodges-Lehmann test. Within-group differences for those variables are analyzed with Wilcoxon Signed Ranks Test.

Significance of between group differences in all variables except for glycated hemoglobin, weight and body mass index was analyzed by the independent samples T-test. Within group differences was analyzed by the paired samples t-test. CI indicates confidence interval. The significance level was set at $P < 0.05$.

For peer review only

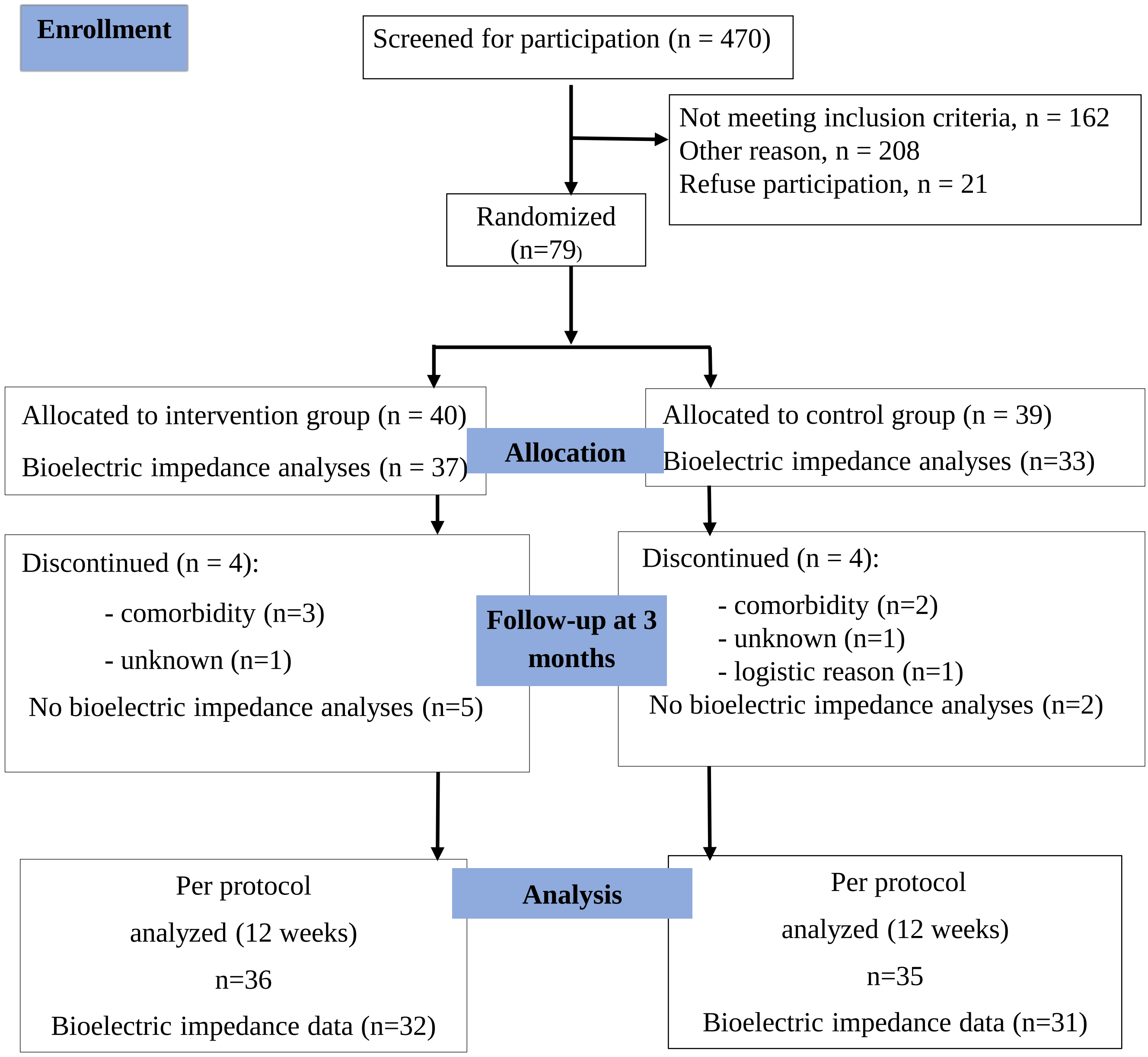
Enrollment
Screened for participation (n = 470)
Not meeting inclusion criteria, n = 162
Other reason, n = 208
Refuse participation, n = 21
Randomized
(n=79)
Allocated to intervention group (n = 40)
Bioelectric impedance analyses (n = 37)
Allocation
Allocated to control group (n = 39)
Bioelectric impedance analyses (n=33)
Discontinued (n = 4):
- comorbidity (n=3)
- unknown (n=1)
No bioelectric impedance analyses (n=5)
Follow-up at 3 months
Discontinued (n = 4):
- comorbidity (n=2)
- unknown (n=1)
- logistic reason (n=1)
No bioelectric impedance analyses (n=2)
Per protocol
analyzed (12 weeks)
n=36
Bioelectric impedance data (n=32)
Analysis
Per protocol
analyzed (12 weeks)
n=35
Bioelectric impedance data (n=31)

# CONSORT 2010 checklist of information to include when reporting a randomised trial*

| Section/Topic | Item No | Checklist item | Reported on page No |
|---|---|---|---|
| **Title and abstract** | | | |
| | 1a | Identification as a randomised trial in the title | Title page |
| | 1b | Structured summary of trial design, methods, results, and conclusions (for specific guidance see CONSORT for abstracts) | Abstract |
| **Introduction** | | | |
| Background and objectives | 2a | Scientific background and explanation of rationale | p.1 |
| | 2b | Specific objectives or hypotheses | p.2 |
| **Methods** | | | |
| Trial design | 3a | Description of trial design (such as parallel, factorial) including allocation ratio | p. 2 |
| | 3b | Important changes to methods after trial commencement (such as eligibility criteria), with reasons | N/A |
| Participants | 4a | Eligibility criteria for participants | p. 2 |
| | 4b | Settings and locations where the data were collected | p. 2 |
| Interventions | 5 | The interventions for each group with sufficient details to allow replication, including how and when they were actually administered | p. 6 |
| Outcomes | 6a | Completely defined pre-specified primary and secondary outcome measures, including how and when they were assessed | p. 3-6 |
| | 6b | Any changes to trial outcomes after the trial commenced, with reasons | N/A |
| Sample size | 7a | How sample size was determined | p. 6 + previous article (p. 2) |
| | 7b | When applicable, explanation of any interim analyses and stopping guidelines | N/A |
| Randomisation: | | | |
| Sequence generation | 8a | Method used to generate the random allocation sequence | p. 3 |
| | 8b | Type of randomisation; details of any restriction (such as blocking and block size) | p. 3 |
| Allocation concealment mechanism | 9 | Mechanism used to implement the random allocation sequence (such as sequentially numbered containers), describing any steps taken to conceal the sequence until interventions were assigned | p. 3 (previous article , p. 2) |
| Implementation | 10 | Who generated the random allocation sequence, who enrolled participants, and who assigned participants to | p. 3 |

For peer review only

| | | | |
|---|---|---|---|
| | | interventions | |
| Blinding | 11a | If done, who was blinded after assignment to interventions (for example, participants, care providers, those assessing outcomes) and how | Previous article, p. 2 |
| | 11b | If relevant, description of the similarity of interventions | N/A |
| Statistical methods | 12a | Statistical methods used to compare groups for primary and secondary outcomes | p. 7 |
| | 12b | Methods for additional analyses, such as subgroup analyses and adjusted analyses | p. 7 |
| **Results** | | | |
| Participant flow (a diagram is strongly recommended) | 13a | For each group, the numbers of participants who were randomly assigned, received intended treatment, and were analysed for the primary outcome | Flow chart |
| | 13b | For each group, losses and exclusions after randomisation, together with reasons | Flow chart |
| Recruitment | 14a | Dates defining the periods of recruitment and follow-up | p. 2 |
| | 14b | Why the trial ended or was stopped | p. 2 |
| Baseline data | 15 | A table showing baseline demographic and clinical characteristics for each group | Table 1 |
| Numbers analysed | 16 | For each group, number of participants (denominator) included in each analysis and whether the analysis was by original assigned groups | Table 1-3 |
| Outcomes and estimation | 17a | For each primary and secondary outcome, results for each group, and the estimated effect size and its precision (such as 95% confidence interval) | Table 3 |
| | 17b | For binary outcomes, presentation of both absolute and relative effect sizes is recommended | N/A |
| Ancillary analyses | 18 | Results of any other analyses performed, including subgroup analyses and adjusted analyses, distinguishing pre-specified from exploratory | N/A |
| Harms | 19 | All important harms or unintended effects in each group (for specific guidance see CONSORT for harms) | p. 9 |
| **Discussion** | | | |
| Limitations | 20 | Trial limitations, addressing sources of potential bias, imprecision, and, if relevant, multiplicity of analyses | p. 11 |
| Generalisability | 21 | Generalisability (external validity, applicability) of the trial findings | Previous article (ref p. 2) |
| Interpretation | 22 | Interpretation consistent with results, balancing benefits and harms, and considering other relevant evidence | p.10-11 |
| **Other information** | | | |
| Registration | 23 | Registration number and name of trial registry | Title page |
| Protocol | 24 | Where the full trial protocol can be accessed, if available | Title page |
| Funding | 25 | Sources of funding and other support (such as supply of drugs), role of funders | p. 13 |

For peer review only



For peer review only - http://bmjopen.bmj.com/site/about/guidelines.xhtml

*We strongly recommend reading this statement in conjunction with the CONSORT 2010 Explanation and Elaboration for important clarifications on all the items. If relevant, we also recommend reading CONSORT extensions for cluster randomised trials, non-inferiority and equivalence trials, non-pharmacological treatments, herbal interventions, and pragmatic trials. Additional extensions are forthcoming: for those and for up to date references relevant to this checklist, see www.consort-statement.org.

For peer review only


For peer review only - http://bmjopen.bmj.com/site/about/guidelines.xhtml
